# Odonate diversity of a highly urbanised region: An annotated checklist of the damselflies and dragonflies (Insecta, Odonata) of Lario and Brianza (Lombardy, N Italy)

**DOI:** 10.3897/BDJ.11.e111358

**Published:** 2023-11-07

**Authors:** Gaia Bazzi, Andrea Galimberti, Claudio Foglini, Luciano Bani, Lionello Bazzi, Piero Bonvicini, Roberto Brembilla, Massimo Brigo, Alberto Cavenaghi, Giuseppe Colombo, Cesare Della Pietà, Carlo Galliani, Ettore Guarnaroli, Nicola Larroux, Alessandro Monti, Valerio Orioli, Francesco Ornaghi, Nicola Pilon, Giuliana Pirotta, Giovanni Radaelli, Giulia Tessa, Giacomo Assandri

**Affiliations:** 1 Area per l’Avifauna Migratrice (BIO-AVM), Istituto Superiore per la Protezione e la Ricerca Ambientale (ISPRA), Ozzano Emilia, Italy Area per l’Avifauna Migratrice (BIO-AVM), Istituto Superiore per la Protezione e la Ricerca Ambientale (ISPRA) Ozzano Emilia Italy; 2 Università degli Studi di Milano-Bicocca, Dipartimento di Biotecnologie e Bioscienze, Milano, Italy Università degli Studi di Milano-Bicocca, Dipartimento di Biotecnologie e Bioscienze Milano Italy; 3 National Biodiversity Future Center, Palermo, Italy National Biodiversity Future Center Palermo Italy; 4 Via L. B. Alberti 8/A, Cinisello Balsamo (MI), Italy Via L. B. Alberti 8/A Cinisello Balsamo (MI) Italy; 5 Università degli Studi di Milano-Bicocca, Dipartimento di Scienze dell'Ambiente e della Terra, Milano, Italy Università degli Studi di Milano-Bicocca, Dipartimento di Scienze dell'Ambiente e della Terra Milano Italy; 6 World Biodiversity Association onlus c/o NAT LAB Forte Inglese, Portoferraio (LV), Italy World Biodiversity Association onlus c/o NAT LAB Forte Inglese Portoferraio (LV) Italy; 7 Centro Ricerche Ornitologiche Scanagatta, Varenna (LC), Italy Centro Ricerche Ornitologiche Scanagatta Varenna (LC) Italy; 8 Odonata.it - Società Italiana per lo Studio e la Conservazione delle Libellula (ODV), Perugia, Italy Odonata.it - Società Italiana per lo Studio e la Conservazione delle Libellula (ODV) Perugia Italy; 9 Via Statale 77ter, Merate (LC), Italy Via Statale 77ter Merate (LC) Italy; 10 Via Cherubini 7, Paderno Dugnano (MI), Italy Via Cherubini 7 Paderno Dugnano (MI) Italy; 11 Odonata.it - Società Italiana per lo Studio e la Conservazione delle Libellule (ODV), Perugia, Italy Odonata.it - Società Italiana per lo Studio e la Conservazione delle Libellule (ODV) Perugia Italy; 12 Gruppo Insubrico di Ornitologia, Clivio (VA), Italy Gruppo Insubrico di Ornitologia Clivio (VA) Italy; 13 Studio Tu.G.A (Tutela e Gestione Ambientale), Rovello Porro (CO), Italy Studio Tu.G.A (Tutela e Gestione Ambientale) Rovello Porro (CO) Italy; 14 Elitron, Milano, Italy Elitron Milano Italy; 15 Via Salerno 12, Lecco, Italy Via Salerno 12 Lecco Italy; 16 Museo Civico di Storia Naturale di Morbegno, Morbegno (SO), Italy Museo Civico di Storia Naturale di Morbegno Morbegno (SO) Italy; 17 Università di Torino, Dipartimento di Scienze della Vita e Biologia dei Sistemi, Torino, Italy Università di Torino, Dipartimento di Scienze della Vita e Biologia dei Sistemi Torino Italy

**Keywords:** Como, Lecco Monza and Brianza, Odonatofauna, urbanisation

## Abstract

**Background:**

Given their sensitivity to environmental alterations, odonates act as reliable bioindicators to assess the effects of changes in freshwater ecosystems and associated terrestrial habitats. The region comprised between Lario and Brianza (Provinces of Como, Lecco and Monza and Brianza - Lombardy, N Italy) is one of the most urbanised of the Italian peninsula and large parts of its territory have been heavily altered, especially at low elevation. Despite this pervasive anthropogenisation, the area is still characterised by a considerable variety of freshwater habitats, possibly harbouring rich odonate communities, which, however, have been never thoroughly investigated. This study aimed to produce the first commented checklist of the Odonata of this region, accompanied by distribution maps.

**New information:**

The work is based on 12,093 records spanning from 1981 and 2022, derived from literature (289), revision of collections (42), citizen-science projects (1249) and unpublished data from the authors and their collaborators (10,513). Overall, fifty-five species occur - or occurred in the past - in the study area (20 Zygoptera and 35 Anisoptera). One species, *Erythrommanajas*, was confirmed exclusively before 1978, while seven species (*Lestesbarbarus*, *Coenagrionscitulum*, *Aeshnaaffinis*, *Anaxephippiger*, *Somatochloraarctica*, *Sympetrummeridionale* and *Trithemisannulata*) have been recorded only after 2000. Records referring to *Chalcolestesparvidens* and *Sympetrumflaveolum* were considered questionable and excluded from the checklist. A list of species for each protected site is additionally provided. This work highlighted the importance for odonates of Lario and Brianza Regions from a national perspective, in particular for species of conservation priority/interest, such as *Sympecmapaedisca*, *Oxygastracurtisii* and *Sympetrumdepressiusculum*.

## Introduction

In the last few years, the evidence of a new environmental crisis has piled up with insects declining at an alarming rate, in terms of biomass, abundance and range (reviewed in Sánchez-Bayo and Wyckhuys (2019), Sánchez‐Bayo and Wyckhuys (2020), Wagner et al. (2021)). Such a phenomenon involves both rare and formerly common taxa and has prominent consequences on entire ecosystems worldwide (Dirzo et al. 2014, Soliveres et al. 2016, Fanin et al. 2017, Sánchez-Bayo and Wyckhuys 2019). However, some key aspects of this crisis are still unknown (Hallmann et al. 2017, Wagner et al. 2021). In this light, faunistic research, trying to fill the so-called ‘Wallacean shortfall’, is gaining relevance as a primary source of information on the current and past presence and distribution of species (Lomolino 2004, Hortal et al. 2015).

Widespread and rather easy to detect and identify, damselflies and dragonflies (Odonata) are amongst the most studied insect taxa (Kalkman et al. 2018). Odonate assemblages respond rapidly to environmental change, making them reliable sentinels of aquatic habitat alterations and indicators of the wider freshwater communities (Corbet 1999, Simaika and Samways 2011, Assandri and Bazzi 2022). Moreover, as adult odonate need resource-rich terrestrial habitats around waterbodies and watercourses, they are also sensitive to changes in landscape composition and configuration, such as urbanisation and agricultural intensification (Nagy et al. 2019 and references therein; Smith et al. 2022).

Given the odonatological tradition and the increased interest raised by this taxon in the last ca. 10 years, nowadays, we have a rather good knowledge of the Italian odonatofauna (Riservato et al. 2014a, La Porta et al. 2023). However, despite the constant monitoring being central to identifying possible anthropogenic impacts and assessing the results of conservation actions, not all the Italian regions have been explored thoroughly in terms of their odonate diversity.

The region between Lake Como (Lario) and Brianza (Lombardy, northern Italy) is one of the most urbanised and densely populated regions of the Country; this determines that virtually every habitat of the region has been altered, especially in the lowland and hills, with important consequences on biodiversity, especially for the avian fauna (Bazzi et al. 2020). However, the region still harbours a remarkable abundance and diversity of freshwater habitats (Siesa 2011) potentially suitable to host rich dragonfly assemblages, but historical studies and records from this area are surprisingly scant, until 2006 at least.

The first odonatological records for Lario and Brianza are due to Vandelli (1763), who, during his naturalistic exploration of the Duchy of Milano, reported five species (*Coenagrionpuella*, *Calopteryxvirgo*, *Corduliaaenea*, *Gomphusvulgatissimus* and *Libelluladepressa*) for the area. Subsequently, with the exclusion of a handful of records cited by Pirotta 1878 and Pirotta 1879, some odonatological reports are found in Morton (1926). He explored the northern part of Lake Como and described the wet habitats he encountered, allowing us, a hundred year later, to understand the impact of the anthropogenic transformations which affected the area. In the subsequent years, a very limited number of records related to rare or uncommon species was dispersed in a few publications (i.e. Nielsen and Conci 1951, Galletti 1968, Capra and Galletti 1978, Balestrazzi and Bucciarelli 1975). Without any doubt, the main contribution to the fauna of the area was given in the revision of the Lombardy odonatofauna by Balestrazzi and Pavesi (2008), who listed the most relevant records in a period comprised between 1930 and 2006, encompassing material from public (Museo di Storia Naturale di Milano and Museo Civico di Storia Naturale di Morbegno) and private collections, including those of several of the most pre-eminent odonatologists of their time (e.g. Cesare Conci, Cesare Nielsen, Italo Bucciarelli and the authors themselves). In their seminal contribution on the dragonflies of Canton Ticino, the Swiss entomologists, De Marmels and Schiess (1978), mentioned some records also for several neighbouring areas, including the Como Province. In the last few years, with the advent of a handful of citizen-science platforms and the spread of interest towards odonates amongst amateurs, the odonatological knowledge of the area has steadily increased.

Thus, the aim of this study was to organise and review the current knowledge of the Odonata of Lario and Brianza in a commented checklist accompanied by distribution maps updated to 2022. The work is based on a database that includes a critical review of the literature, scientific collections and - mainly - recent unpublished data, collected thanks to the collaboration of more than 220 observers and validated by experts.

## Materials and methods

### Study area

**Figure 1.**
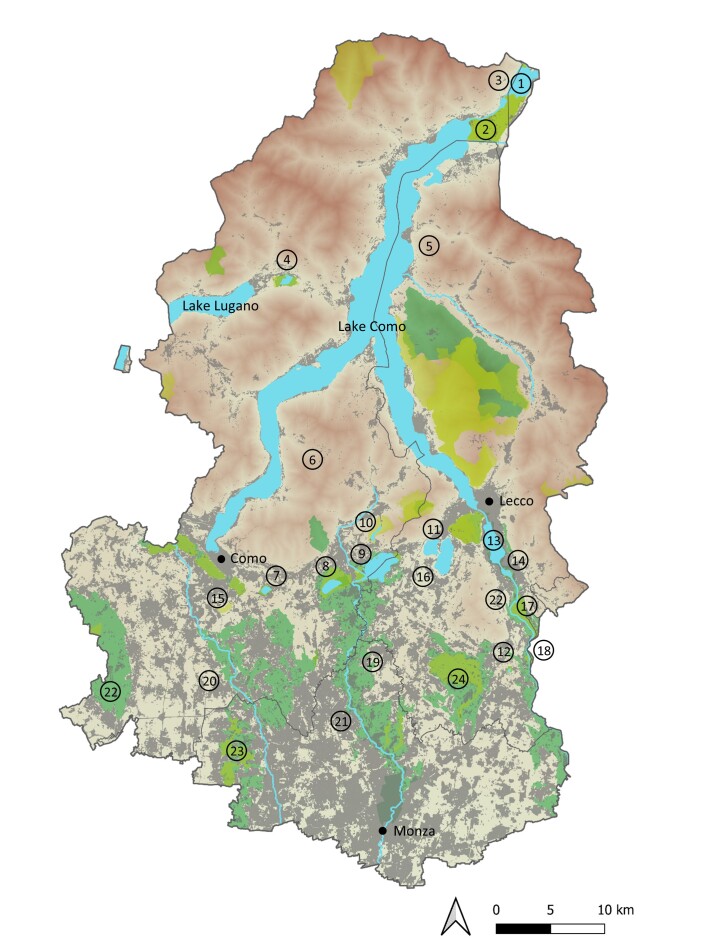
Map of the study area. The main freshwater sites and the three main cities are shown. Regional parks and Natural Reserves are shown in dark green, Natura 2000 sites in yellow. Urban areas and infrastructures are shown in grey. Main localities cited in the text: 1) Lake Mezzola CO-SO; 2) Pian di Spagna CO-SO and Erbiola wetland LC; 3) Laghetti della Peschiera CO; 4) Lake Piano CO; 5) Camaggiore fen LC; 6) Piani di Nesso-Pian del Tivano fen CO; 7) Lake Montorfano CO; 8) Lake Alserio CO; 9) Lake Pusiano CO-LC; 10) Lake Segrino CO; 11) Lake Annone LC; 12) Lake Sartirana LC; 13) Lake Garlate LC; 14) Lake Olginate LC; 15) Oasi del Bassone-Torbiere di Albate, CO; 16) La Poncia LC; 17) Palude di Brivio LC; 18) Toffo-Oasi dell’Alberone LC-BG; 19) Cariggi, Renate MB; 20) Seveso River CO-MB; 21) Lambro River CO-LC-MB; Adda River LC-MB; 22) Parco Pineta di Appiano Gentile e Tradate CO; and 23) Parco delle Groane CO-MB; 24) Parco di Montevecchia e della Valle del Curone LC.

The study area covers the Provinces of Como, Lecco (including the municipality of Torre de’ Busi, which moved to the Bergamo Province in 2018) and Monza and Brianza in central-northern Lombardy (N Italy) (Fig. 1). Additionally, three small sites, i.e. Lake Mezzola (between Como and Sondrio Provinces), Toffo-Oasi dell’Alberone (between Lecco and Bergamo Provinces) and a newly-created wetland between Mozzate and Cislago (Como and Varese Provinces, respectively), which straddle the border of the three provinces, were included due to their geographical and environmental continuity with three important wetlands of the study area. Overall, the investigated region extends from the upper Po Plain to the Central Alps (Lepontine and Orobics) for a total surface of 2,510 km^2^ and an altitudinal range comprising between 137 and 2609 m a.s.l. reached by Mount Legnone, the highest peak in the range. Brianza is heavily anthropogenised, with most of its territory covered by urban areas and infrastructures and very few natural or semi-natural remnants. Indeed, Monza and Brianza is the second most densely populated province in Italy, accounting for 2,150 inhabitants/km^2^, while Como and Lecco rank 10^th^ and 13^th^, with 466 and 412 inhabitants/km^2^, respectively (dati.istat.it).

Located in the middle of the Italian Lake District, the study area shows a high environmental heterogeneity, with a variety of lentic and lotic habitats. The northern part of the region is mountainous. The Alps and the Pre-Alps are cut from north to south by the valley of Lake Como and in the west by that of Lake Lugano, two large fluvial-glacial oligotrophic lakes (146 and 49 km^2^, respectively) characterised by deep, rather cold waters and rocky shores. This area hosts a few lowland wetland systems; however, amongst them, Pian di Spagna-Lake Mezzola (comprising also the smaller Erbiola wetland) and Lake Piano are those of the greatest naturalistic interest. Other minor wetlands, for example, those along the northern shores of Lake Como, have been reclaimed and then progressively urbanised in the past and are now completely lost (Morton 1926). All the other aquatic habitats in the northern part are located at medium or high elevation and are mostly represented by small ponds, watering pools and mountain streams. Two fens, located in Camaggiore and Piani di Nesso-Pian del Tivano (ca. 1160 and 950 m a.s.l., respectively), deserve to be mentioned as being rare ecosystems in this region.

South of the Alps and the Pre-Alps lies a hilly belt that gradually slopes down to the upper Po Plain. This portion of the study area, known as Brianza, is crossed by a few rivers, the major from west to east being the Seveso, Lambro and Adda and is scattered with small- and medium-sized lakes. From west to east are found Lake Montorfano, Lake Alserio, Lake Pusiano, Lake Segrino, Lake Annone and Lake Sartirana, characterised by rather shallow and eutrophic waters and extended floating plant communities and reed-beds and Lake Garlate and Lake Olginate, which originate from expansions of the River Adda south of the City of Lecco. Around these lakes, along some of the main rivers and in the depressions between the hills lie small remnants of ancient peatlands, marshlands, hygrophilous woods and wet meadows, crossed by a network of natural and artificial channels and ditches. Of particular interest are Oasi del Bassone-Torbiere di Albate, La Poncia wetland, Palude di Brivio, Toffo-Oasi dell’Alberone and Cariggi. Other important lentic habitats consist of ephemeral wetlands within heathlands, quarry lakes and ponds created as part of environmental restorations.

### Data source and curation

**Figure 2.**
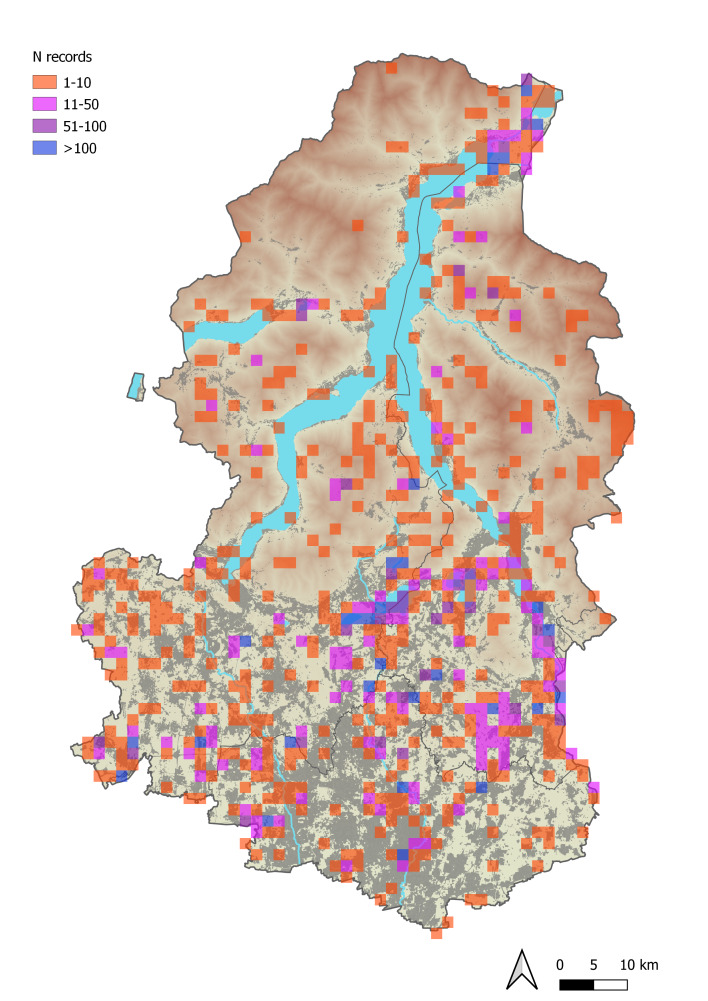
Spatial distribution of odonate records in the study area. Cell colour indicates the number of records based on a 1 km grid.

This checklist is based on 12,093 records collected between 1851 and 2022, although most of them (11,858) refer to recent years (2007-2022). Overall, data were derived from literature, scientific collections, citizen-science repositories and, in the great majority, from authors’ and collaborators’ field campaigns. Literature records were 289 and derived from 10 documents, in which at least one new record was present. Data exclusively referred to specimens from scientific collections totalled 42, all conserved in the collection of the Museo Civico di Storia Naturale di Morbegno. Other records deriving from collections were published by Balestrazzi and Pavesi (2008) and, thus, considered as literature. Collection and literature records were scrutinised, validated and assigned to discrete localities (areas with defined boundaries, clearly distinguishable from aerial photographs) and georeferenced. A total of 1249 records were derived from citizen-science projects; from www.inaturalist.org (accessed 31/12/2022), we obtained 1178 records and from www.observation.org (accessed 18/12/2022) 71. For citizen-science records we only retained data revised by ourselves, with photographs and excluded those with very inaccurate coordinates (> 10 km). The remaining 10,513 records were unpublished and derived from recent (most of them later than 2006) field surveys by the authors and their collaborators. The spatial coverage of the dataset is shown in Fig. 2. Site elevation, when not specifically reported, was obtained, based on coordinates from a DTM 20 x 20 m grid. The entire dataset, on which this checklist is based, is accessible at https://ipt.pensoft.net/resource.do?r=odonate_lccomb.

We provide a list of the species recorded in the study area. For all species, we give a distribution map (Suppl. material 1), distinguishing records before 2000 and subsequent (for consistency with the Provisional Atlas of Italian dragonflies and damselflies; Riservato et al. (2014a)) and a brief account including comments on distribution, flight period (referred to the “post-2000” period), habitat and conservation issues. Emphasis is given to records which are important from an Italian perspective or a conservation point of view. The lists follow the nomenclature and systematic order of the Italian check-list of odonates (La Porta et al. 2023).

Additionally, to favour conservation and monitoring activities, we provide species lists for each protected area found in the study area according to the national/local legislation and the Natura 2000 network, again distinguishing occurrence before and after 2000 (Suppl. materials 2, 3).

### Legend of the symbols used in the check-list

**Native status**

For each species treated in the checklist, we provided details on the status in the area:

*: species recorded only before 2000;
- **R**: reproduction confirmed in the study area;
- **R**?: reproduction possible, although not confirmed;
- **Re**: reproduction confirmed/possible before 2000, now extinct as a reproductive species;
- **NR**: reproduction not confirmed in the study area;
- **EX**: species excluded from the fauna of the study area.

**Conservation status**

For each species treated in the checklist, we provided details on:

- **hd**: Habitats Directive (Council Directive 92/43/EEC of 21 May 1992 on the conservation of natural habitats and of wild fauna and flora). The Annexes (II and/or IV) in which the species is listed are specified;
- **erl**: European Red List of Dragonflies (Kalkman et al. 2010);
- **irl**: Italian Red List of Dragonflies (Riservato et al. 2014b).

For both the Red Lists, the IUCN category is provided following these abbreviations:

- CR: Critically endangered;
- EN: Endangered;
- VU: Vulnerable;
- NT: Near threatened;
- LC: Least concern.

## Checklists

### Checklist of the Odonata of Lario and Brianza

#### Calopteryx splendens

(Harris, 1780)

315FC814-DE51-59CC-9860-1112D616A9F7

##### Native status

R

##### Conservation status

erl: LC; irl: LC

##### Notes

Flight period: III April - III October

The species is fairly widespread in the study area, up to 800 m a.s.l. It occurs in a variety of lotic habitats, though it seems more abundant along the course of the main rivers and streams, such as Adda (both north and south of Lake Como), Molgora LC-MB, Lambro, Seveso and Lura CO, also in extensively urbanised areas.

#### Calopteryx virgo

(Linnaeus, 1758)

29ADA66E-A133-5DFE-9B91-D13DAF328CFB

##### Native status

R

##### Conservation status

erl: LC; irl: LC

##### Notes

Flight period: I May - II November

Compared with the congeneric *C.splendens*, this species is more frequent at the foothills and the lower mountain slopes, up to ca. 900 m a.s.l., whereas it is scarce in the lowlands. This is explained by its stricter ecological requirements, as it reproduces in small, well-oxygenated and fresh lotic habitats rich in aquatic vegetation and with a structured riparian belt, often in wooded contexts. Being more stenotopic, *C.virgo* could be more susceptible to anthropogenic environmental alterations. For this reason, the Province of Monza and Brianza, which is predominantly flat and largely urbanised, is nowadays only marginally affected by its presence. A record of two individuals on 18.11.2021 at Lake Alserio (P. Bonvicini, obs.) should be considered as remarkably late for the species.

#### Sympecma fusca

(Vander Linden, 1820)

716CCD47-3CE6-5CF1-8B75-F5CACF224AB5

##### Native status

R

##### Conservation status

erl: LC; irl: LC

##### Notes

Flight period: I February - I November

Based on historic data, this species appears to have been widely distributed in the lowlands and hills of the whole study area, in a variety of habitats comprising permanent and ephemeral marshlands up to ca. 400 m a.s.l. Now it holds a rather patchy distribution, possibly suggesting a local decline, with a few strongholds mainly concentrated at Pian di Spagna-Lake Mezzola and the heathlands of CO-MB. Available data are referred to the whole life cycle, including winter, with the exception of December and January, when the species is less easily detectable.

#### Sympecma paedisca

(Brauer, 1877)

0DF25BC5-D04C-5B4C-AD15-743183884566

##### Native status

R?

##### Conservation status

hd: II; erl: LC; irl: CR

##### Notes

Flight period: II March - III May and I November

**Figure 3.**
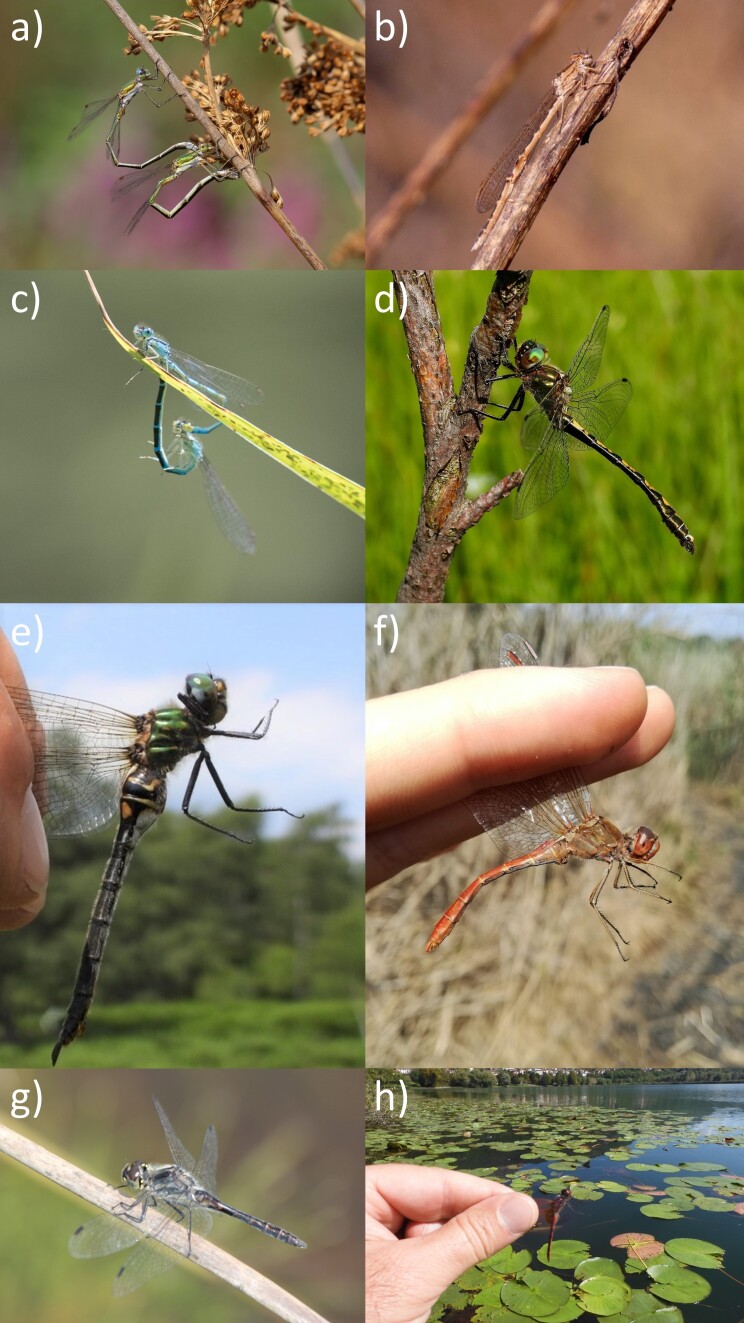
Relevant species/records of Odonata in Lario and Brianza Regions (N Italy); a) *Lestesvirens*, Parco delle Groane; b) *Sympecmapaedisca*, Parco della Pineta di Appiano Gentile e Tradate; c) *Coenagrionscitulum*, Parco di Montevecchia e della Valle del Curone; d) *Oxygastracurtisii*, Lake Alserio; e) *Somatochloraarctica*, Camaggiore fen; f) *Sympetrummeridionale*, Pusiano Lake; g) *Sympetrumdanae*, Monte Muggio, Casargo; h) *Trithemisannulata*, Piano Lake. Photos by G. Bazzi, N. Larroux, C. Della Pietà, A. Cavenaghi, P. Bonvicini and N. Pilon.

This species was reported historically for Calolziocorte LC (31.05.1935, Nielsen and Conci (1951); the exact site was not specified, but it was likely the Palude di Brivio), where, considering the date, probably it used to breed. Subsequently, no further evidence emerged until it was newly reported from two localities in the southern part of Parco della Pineta di Appiano Gentile e Tradate in the 2020-2021 winter (N. Larrorux obs., Fig. 3b). This area is characterised by heathland relicts similar to those that harbour the core of the Italian population of the species in Piedmont (Battisti and Pavesi 2017). This recently-discovered site represents the new eastern limit of the species’ range in Italy, as it is deemed extinct in Trentino (Assandri 2019). Given the conservation importance of this declining species, further research and conservation actions should be undertaken at Parco Pineta. Specifically, putative breeding sites should be surveyed and, when confirmed, granted adequate protection.

#### Chalcolestes viridis

(Vander Linden, 1820)

0F686130-0950-55A1-B3B7-380AFAB11136

##### Native status

R

##### Conservation status

erl: LC; irl: LC

##### Notes

Flight period: II June - II November

Occurs from the lowlands to the hills, up to 450 m a.s.l., in a wide variety of lentic and lotic habitats of the southern part of the study area. An isolated population is found at Pian di Spagna-Lake Mezzola.

#### Lestes barbarus

(Fabricius, 1798)

BC0809D8-245A-5501-BB11-19384DCBA460

##### Native status

NR

##### Conservation status

erl: LC; irl: LC

##### Notes

It is known for only one record, a single individual observed on 06/09/2012 at Calendone pond, Merate LC within the Parco Regionale di Montevecchia e della Valle del Curone (V. Orioli obs.). In Italy, the species has stable populations mainly in the centre and south, but it is highly dispersive and undertakes regular movements towards the north (Riservato et al. 2014a, Assandri 2019); this helps to explain its occasional occurrence in the study area.

#### Lestes sponsa

(Hansemann, 1823)

65C119D2-1D66-5E18-9D1A-DACD8C78B4A5

##### Native status

R

##### Conservation status

erl: LC; irl: LC

##### Notes

Flight period: I June - III September

Species localised in the study area, it has been reported only at Pian di Spagna-Lake Mezzola, Parco delle Groane CO-MB, Parco della Pineta di Appiano Gentile e Tradate CO and Palude di Brivio. *L.sponsa* is mainly found in two habitat typologies: lowland ephemeral wetlands within heathlands and wet meadows, up to ca. 280 m a.s.l. Although there is no clear evidence due to the few historical records available, it is likely that this species, like others with similar ecological preferences, decreased in the last century, as reported in neighbouring regions (Wildermuth et al. 2005, Assandri 2020).

#### Lestes virens

(Charpentier, 1825)

BBA8EA4B-9E77-5020-8816-AC7F577D01A9

##### Native status

R

##### Conservation status

erl: LC; irl: LC

##### Notes

Flight period: I June - I September

The species, which is tied to ephemeral habitats characterised by temporary waters and a high cover of aquatic plants, in the study area is found solely in the temporary wetlands within the heathlands of Parco delle Groane, up to ca. 280 m a.s.l. (Fig. 3a).

#### Platycnemis pennipes

(Pallas, 1771)

E57EF5D7-2DCC-50A9-9EE1-7F8971862F54

##### Native status

R

##### Conservation status

erl: LC; irl: LC

##### Notes

Flight period: II April - III September

In the study area, the species is found mainly at low elevation up to 940 m a.s.l. in a variety of lotic and lentic habitats; the species is, in fact, known to be a generalist and tolerant to anthropogenic alterations. Occurrence records are mostly concentrated in the southern part of the study area, even if small populations occur further north at Pian di Spagna-Lake Mezzola, Lake Piano and along the western coast of Lake Como. It is surprisingly absent from the western portion of Como Province, where other species with similar ecological requirements occur.

#### Ischnura elegans

(Vander Linden, 1820)

DF819A34-A114-5FB5-9F8C-7B9FC1807EBC

##### Native status

R

##### Conservation status

erl: LC; irl: LC

##### Notes

Flight period: III March - III October

Common and widespread in the study area, it occurs mainly in the lowlands and on the hills (though some observations refer to higher elevation, up to more than 1500 m a.s.l.), in a wide range of lentic and lotic habitats, also at heavily urbanised sites.

#### Ischnura pumilio

(Charpentier, 1825)

E6EE5782-BCDB-5A89-A789-82291738A981

##### Native status

R

##### Conservation status

erl: LC; irl: LC

##### Notes

Flight period: III April - III September

A pioneer species, in the study area is widespread in newly-established ponds, wet meadows and temporary wet habitats which are prone to drying out, generally avoiding mature wetlands. It occurs from the lowland up to ca. 1550 m a.s.l. and could be sometimes found far away from suitable breeding sites and in rather urbanised areas.

#### Enallagma cyathigerum

(Charpentier, 1840)

5B484034-DF17-5C0E-A1EA-D8465E9F62DD

##### Native status

R

##### Conservation status

erl: LC; irl: LC

##### Notes

Flight period: II April - I October

The species in the study area is found in three main habitats: 1) medium or large lakes with open waters (e.g. Lakes Como, Pusiano and Annone); 2) large and clean rivers, in particular the Adda and Mera CO, which are the only rivers at which the species is found in the study area; and 3) mountain watering or firefighting ponds up to 1750 m a.s.l. It is surprisingly absent at Lake Lugano and lacks recent confirmation at Lake Alserio, whereas its absence in the highly urbanised Monza and Brianza Province is not surprising.

#### Pyrrhosoma nymphula

(Sulzer, 1776)

167FB9FB-4636-5104-9455-C1242412F80D

##### Native status

R

##### Conservation status

erl: LC; irl: LC

##### Notes

Flight period: I April - III July

The species is rather localised in the study area, occurring in the belt between the south of Lake Como and north to the heavily urbanised area of the Monza and Brianza. There, it is mainly found in mature and well-preserved marshlands and along small streams, covered by rich aquatic vegetation, up to ca. 400 m a.s.l. Historical records at higher elevation (i.e. between ca. 400 m a.s.l. and ca. 1000 m a.s.l., at Piani di Nesso and Lake Segrino; Balestrazzi and Pavesi (2008)) were not confirmed in recent years.

#### Coenagrion puella

(Linnaeus, 1758)

AF53314C-21A3-53F5-BA26-5D7189F37FF1

##### Native status

R

##### Conservation status

erl: LC; irl: LC

##### Notes

Flight period: III March - I August

Common and widespread throughout the whole study area, it occurs in marshlands, lakes, ponds, wet meadows, rivers and ditches, from the lowlands to medium elevation in the mountains, with records up to 1562 m a.s.l. The species is scarcer in the central and eastern Monza and Brianza Province, possibly due to massive urbanisation that compromised its habitats.

#### Coenagrion pulchellum

(Vander Linden, 1825)

18E1EEEA-32F3-58B9-B14F-24F9143B18C4

##### Native status

R

##### Conservation status

erl: LC; irl: NT

##### Notes

Flight period: II April - I August

Localised in the study area; it is tied to well-preserved marshlands with rich aquatic and riparian vegetation, mainly at low elevation (max. elevation recorded: 597 m a.s.l.). Specifically, it is found at Pian di Spagna-Lake Mezzola, Laghetti della Peschiera, Lake Piano, Lake Segrino, Lake Alserio, Lake Montorfano, Lake Pusiano, two sites within the Parco Regionale di Montevecchia e della Valle del Curone LC and Toffo-Oasi dell’Alberone. Its occurrence at Lakes Annone and Sartirana, recorded in 1948 and 1977, respectively, was not confirmed in recent years; however, further investigations of these sites are needed to confirm its local extinction.

#### Coenagrion scitulum

(Rambur, 1842)

C591CDD8-6B20-5286-A33B-0220A8BAB081

##### Native status

R

##### Conservation status

erl: LC; irl: LC

##### Notes

Flight period: III May - III June

A southern species, it is known to colonise newly-created suitable habitats, i.e. lentic or slow-flowing waters rich in aquatic vegetation also in response to the increasing temperature determined by climate warming (Boudot and Kalkman 2015). In the study area, *C.scitulum* was first discovered in 2019, when a population was found at a small artificial pond at the south-eastern border of the Como Province, between Mozzate and Cislago (N. Larroux obs.). However, the occurrence of this population was never confirmed in the following years. Subsequently, in 2020, a mating pair was observed at Calendone Pond in the Parco di Montevecchia e della Valle del Curone, which had just undergone restoration (C. Della Pietà obs., *Fig. 3*c). Additionally, in this case, no further observation followed the first and a prolonged drought in the summer of 2022 caused the pond to completely dry out, with likely strong impacts on the entire odonate community.

#### Erythromma lindenii

(Selys 1840)

F1616139-1C23-5223-B1BC-80312AF64F6C

##### Native status

R

##### Conservation status

erl: LC; irl: LC

##### Notes

Flight period: II May - II September

In the study area, the species is mainly localised along the Adda River and at medium-sized lakes, warm and rich in aquatic vegetation, such as Lakes Annone, Segrino, Piano, Pusiano and Montorfano, where recent confirmation is lacking. It also occurs at quarry lakes (Cave di Baggero, Merone CO) and it is surprisingly absent at Lakes Como and Lugano.

#### Erythromma najas

(Hansemann, 1823)

43CEC5C7-8089-552D-85F6-83331E159944

##### Native status

* Re

##### Conservation status

erl: LC; irl: EN

##### Notes

Occurring at least until the second half of the 1970s at Lake Piano and Lake Annone (Balestrazzi and Bucciarelli 1975, De Marmels and Schiess 1978, Balestrazzi and Pavesi 2008), to the best of our knowledge, the species is now extinct from the study area. The only record accompanied by a complete date refers to 02.06.1978.

#### Erythromma viridulum

(Charpentier, 1840)

83026793-83C8-5EC5-9FE3-6D961FAA81E3

##### Native status

R

##### Conservation status

erl: LC; irl: LC

##### Notes

Flight period: III May - II October

In the study area, the species occurs in rather few low-elevation localities, up to ca. 400 m a.s.l., where lakes, channels or slow-flowing rivers with warm waters and rich floating aquatic vegetation are found. Its occurrence at Lake Piano and Lake Sartirana, where the species was observed in 1977 and 1958, respectively (De Marmels and Schiess 1978, Balestrazzi and Pavesi 2008), has not been confirmed in recent years.

#### Ceriagrion tenellum

(de Villers, 1789)

D52EDE23-1483-579B-87CF-9B3491821B60

##### Native status

R

##### Conservation status

erl: LC; irl: LC

##### Notes

Flight period: I June - I October

Rather localised, in the study area, it occurs at well-preserved mature marshes and peatlands, for example, those associated with small- and medium-sized lakes (e.g. Alserio, Pusiano, Segrino, Piano and Annone) and along streams and rivers. It is a thermophilic species; it is found up to ca. 450 a.s.l. Only a single record at higher elevation (01.08.2013, Laghetto di Monte Tellero, Ponna Superiore CO, 1116 m a.s.l.; N. Pilon obs.).

#### Aeshna affinis

Vander Linden, 1820

7345B904-623F-51B2-9C19-4CFEFE07948B

##### Native status

R

##### Conservation status

erl: LC; irl: LC

##### Notes

Flight period: I July - III August

Rather localised, it has been recorded at a few sites: Pian di Spagna-Lake Mezzola, Parco della Pineta di Appiano Gentile e Tradate, Parco delle Groane, northern part of the Parco della Valle del Lambro CO-LC-MB and Parco Regionale di Montevecchia e della Valle del Curone. It occurs mainly at wet meadows and ephemeral wetlands, such as those found in heathlands, at low elevation (max. 380 m a.s.l.). The lack of historical data could suggest a recent colonisation of the area.

#### Aeshna cyanea

(Müller, 1764)

9BF52208-39EE-57AB-A1CB-51AF8E8D43A5

##### Native status

R

##### Conservation status

erl: LC; irl: LC

##### Notes

Flight period: II June - II November

A generalist species, it is widespread in all the sectors of the study area, from the lowlands to the mid-mountain, with records up to ca. 1700 m a.s.l. It occurs in a wide array of habitats, although it is rarely abundant, with a predilection for small, partially shaded ponds or ditches.

#### Aeshna isoceles

(Müller, 1767)

8FD2066D-F157-5393-9EED-9450D472204B

##### Native status

R

##### Conservation status

erl: LC; irl: LC

##### Notes

Flight period: II April - II August

Rather widespread in the study area, wherever suitable habitats occur. It is found primarily in wetlands with at least a small extension of reed-beds, up to 800 m a.s.l. More localised in the Monza and Brianza Province, likely due to the heavy anthropogenisation of the area.

#### Aeshna juncea

(Linnaeus, 1758)

D37A4861-DB63-5C3D-8948-B996CAC07BDA

##### Native status

R

##### Conservation status

erl: LC; irl: LC

##### Notes

Flight period: II June - I October

The species is found solely in the mountains, between ca. 950 and 1920 m a.s.l., the only exceptions being two historical records from Pian di Spagna at 200 m a.s.l. (one individual on 08.10.1972 and two on 08.10.1988, G. Perego, Museo Civico di Storia Naturale di Morbegno collection, Balestrazzi and Pavesi (2008)), without further recent confirmation. It is absent from the Monza and Brianza Province, which is entirely below 400 m a.s.l.; the low number of observations in the Como Province is likely attributable to a lack of observations. In the study area, the species adapted to small watering ponds with at least a little aquatic or helophytic vegetation.

#### Aeshna mixta

Latreille, 1805

FA86719B-C624-574D-9277-6865B8183A28

##### Native status

R

##### Conservation status

erl: LC; irl: LC

##### Notes

Flight period: I July - III November

The species occurs at low elevation in the study area, mainly up to ca. 370 m a.s.l., except for a handful of records from Triangolo Lariano CO-LC and Laghetti della Peschiera, ranging between ca. 500 and 800 m a.s.l. It occurs primarily at mature and well-structured wetlands, but it is occasionally found also in more degraded areas or at artificial ponds.

#### Anax imperator

Leach in Brewster, 1815

D77D796C-B5DF-5341-897B-C7CE35038B23

##### Native status

R

##### Conservation status

erl: LC; irl: LC

##### Notes

Flight period: I April - I November

Widespread in the study area, it is found from the lowlands to rather high elevations (max. elevation 2078 m a.s.l.), in large and small wetlands, ponds and in the slow-flowing stretches of rivers, streams and ditches, even in highly-urbanised sites.

#### Anax parthenope

(Selys 1839)

4FA092C6-1CCF-5413-AF39-AAC84C81F5DC

##### Native status

R

##### Conservation status

erl: LC; irl: LC

##### Notes

Flight period: I April - III September

Much more localised than the congeneric *A.imperator*, in the study area, the species is found at low-medium elevation, up to ca. 810 m a.s.l. It occurs at a wide array of lentic or slow-flowing waters, such as mature marshlands rich in aquatic and riparian vegetation, but also small artificial ponds in heavily-urbanised contexts; nevertheless, it is found at relatively few sites: Pian di Spagna, Lake Piano, Parco della Pineta di Tradate e Appiano Gentile and surroundings, Lura River, Parco delle Groane, Parco della Valle del Lambro and surroundings, Lake Crezzo, Lake Segrino, Lake Sartirana and the northern part of the Parco Adda. Its apparent demise from Lake Annone could be attributable to a lack of recent information about this area.

#### Anax ephippiger

(Burmeister, 1839)

970D986A-05E7-59E7-A9E8-E859C3C6264F

##### Native status

NR

##### Conservation status

erl: LC; irl: LC

##### Notes

Flight period: III June - II September

The species has been recorded in the study area only since 2018 and most of the records refer to 2019, when a massive invasion of this migrant and pioneer species was recorded in Europe (Manger and De Knijf 2022). Most of the records refer to August-September, in line with the phenology of the species in northern Italy (e.g. Boano et al. (2007)) and no evidence of reproduction in the area has been collected until now.

#### Gomphus vulgatissimus

(Linnaeus, 1758)

2D26657E-8CAD-5CFA-8264-6D5CF3BF67FE

##### Native status

R

##### Conservation status

erl: LC; irl: LC

##### Notes

Flight period: II April - III July

In the study area, this rheophilic species is almost restricted to the course of two rivers, namely Lambro CO-MB, Adda LC and their tributaries, up to ca. 350 m a.s.l. Additionally, the species is found in the Alserio Lake area, where the River Lambro exits the Lake and receives several artificial channels from the Piani d’Erba. Its occurrence at Lago del Segrino has not been confirmed after 1960.

#### Onychogomphus forcipatus

(Linnaeus, 1758)

E14BF496-4895-5D4E-9C64-E9230435097C

##### Native status

R

##### Conservation status

erl: LC; irl: LC

##### Notes

Flight period: III May - I October

It is found along a wide variety of rivers, ditches and lake shores of the study area, even within urban areas and mainly in the absence of riparian vegetation, in the lowlands and valley bottoms. It is one of the very few species breeding in Como Lake. Dispersing individuals have been observed far from their breeding habitats, even in mountains (max. elevation: 1059 m a.s.l.).

#### Cordulegaster bidentata

Selys, 1843

CD3882F5-79FE-5C93-ADD7-39954F5A52DC

##### Native status

R

##### Conservation status

erl: NT; irl: LC

##### Notes

Flight period: III May - II September

The species is widespread in the hilly areas and mountains of the study area, as far south as Parco di Montevecchia e della Valle del Curone, being completely absent from the Monza and Brianza Province. It frequents small, shaded streams and rivulets in forested areas, between ca. 190 and 1510 m a.s.l. Owing to the difficulties of separation from the congeneric *C.boltonii*, the species could have been slightly under-recorded, especially at lower elevations.

#### Cordulegaster boltonii

(Donovan, 1807)

95EAC7DB-D13B-54BC-8907-EAF41046422E

##### Native status

R

##### Conservation status

erl: LC; irl: LC

##### Notes

Flight period: I June - III September

It is generally found at lower elevation than the congeneric *C.bidentata*, although it can reach 1200 m a.s.l. Widespread in the hilly belt of high Brianza, it is rare in the Monza and Brianza province. It occupies small streams and ditches, both in wooded and open areas.

#### Cordulia aenea

(Linnaeus, 1758)

91DA462D-672F-5857-8C36-80D32F814A88

##### Native status

R

##### Conservation status

erl: LC; irl: NT

##### Notes

Flight period: II April - I July

Rather localised in the study area, it is found mostly at mature and well-preserved marshlands up to ca. 800 m a.s.l., in particular, those associated with medium-sized natural lakes, but also to well-conserved marshland along rivers, such as the Toffo-Oasi dell’Alberone along the Adda River. Evidence of reproduction exist also for several small ponds within heathlands and a single exuvia was found in a small artificial pond on the Monte Barro, suggesting that dispersing individual from large populations can occasionally breed in sub-optimal habitats.

#### Somatochlora arctica

(Zetterstedt, 1840)

9F84D789-B885-5938-8A07-DAF8DA0A6FE0

##### Native status

R

##### Conservation status

erl: LC; irl: NT

##### Notes

Flight period: II July - II August

In the study area, the species was discovered in 2021 at the Camaggiore fen, Vendrogno (1160 m a.s.l.; P. Bonvicini obs., Fig. 3e), where a small breeding population was subsequently confirmed to exist. This is likely the only suitable biotope for the species in the three provinces, the closest known sites of presence being in the nearby, but more climatically and environmentally favourable, Orobic Alps (Riservato et al. 2014a).

#### Somatochlora flavomaculata

(Vander Linden, 1825)

7D0B12DD-182C-5BF7-96AA-17F98E221E75

##### Native status

R

##### Conservation status

erl: LC; irl: LC

##### Notes

Flight period: III May - I September

Rather localised in the lowlands and valley bottoms below 400 m a.s.l., it is found mainly in mature and well-preserved marshlands and in the surrounding open areas, where it usually hunts. Data from literature suggest that, in the past, the species was much more widespread; it is possible that urbanisation and habitat alteration led to the disappearance of the species from a few sites, such as north-western Lake Como, where it was historically reported by Morton (1926) and Lake Olginate.

#### Somatochlora metallica

(Vander Linden, 1825)

08C732DE-A840-5C10-9A70-24393CB1F3CB

##### Native status

R

##### Conservation status

erl: LC; irl: LC

##### Notes

Flight period: III May - II October

Widespread in the study area, especially below 700 m a.s.l., with scattered records up to 1200 m a.s.l. It occurs at a large variety of habitats, ranging from wetlands and ponds to slow-flowing stretches of rivers and lake shores and is often found far from suitable breeding sites, also in urban contexts. The apparent absence from Lake Lugano and other sites in the Como Province could be attributed to the limited data available for this area.

#### Oxygastra curtisii

(Dale, 1834)

DA37C991-9D4A-5CB8-B4C7-F644EE038A6C

##### Native status

R

##### Conservation status

hd: II, IV; erl: NT; irl: NT

##### Notes

Flight period: III May - III August

This quite rare and conservation concern species emerged as a rather widespread dragonfly in the study area (Fig. 3d). Breeding populations were confirmed for Lake Lugano, Lake Piano, Lake Como, Lake Alserio (at the origin of the Lambro River) and Lake Segrino and along the course of the Adda River, where the species is abundant. In the Pian di Spagna area, there is only one piece of evidence of an immature individual possibly born in Lake Como or in the River Mera. Maturing individuals are frequently found rather far from breeding areas, often on grassland mountain slopes up to 1200 m a.s.l.

#### Libellula depressa

Linnaeus, 1758

C0104107-5A8F-545A-B3A6-7A674D8C5859

##### Native status

R

##### Conservation status

erl: LC; irl: LC

##### Notes

Flight period: I April - I September

Common and widespread in the study area, from the lowlands to high elevation (max. 1913 m a.s.l.). It is found in a variety of lentic and lotic habitats, often of very small extent and ephemeral. The apparent absence from the northern part of Como Province is likely due to a lack of data for this area.

#### Libellula fulva

Müller, 1764

BF19FC06-FC6F-5D73-90BA-381A721A6C10

##### Native status

R

##### Conservation status

erl: LC; irl: LC

##### Notes

Flight period: III April - II August

The species mostly inhabits low elevation rivers and small- and medium-sized lakes up to ca. 400 m a.s.l., rich in aquatic vegetation and, in particular, reed-beds. Rather localised in the Monza and Brianza Province, where it is found almost exclusively along the River Lambro.

#### Libellula quadrimaculata

Linnaeus, 1758

E7B2003D-CC15-5EB0-8FD1-084C3E9C850A

##### Native status

R

##### Conservation status

erl: LC; irl: LC

##### Notes

Flight period: III April - I September

Widespread in the Como and Lecco Provinces, from the lowlands up to 1900 m a.s.l. Rarer in Monza and Brianza, where it is confined to the Parco delle Groane and its surroundings. It occurs mostly in mature marshlands, small lakes and ponds and watering ponds.

#### Orthetrum albistylum

(Selys, 1848)

8CE8F408-2EF2-5ABE-9201-DB684FD8B380

##### Native status

R

##### Conservation status

erl: LC; irl: LC

##### Notes

Flight period: II May - II September

The species occurs mainly south of Lake Como, where it is common and widespread in small and medium lakes, ponds and ditches up to ca. 400 m a.s.l. An isolated population, which inhabits the northern end of the area (i.e. Pian di Spagna and, notably, Laghetti della Peschiera, at ca. 600 m a.s.l.), seems the result of recent colonisation, as there are no historical records referred to these well-explored sites. This is in line with previous evidence of a northwards expansion of the species in Europe, attributable to climate change (Boudot and Kalkman 2015).

#### Orthetrum brunneum

(Fonscolombe, 1837)

DA6E2773-A89D-5790-9A30-6F3584604FE3

##### Native status

R

##### Conservation status

erl: LC; irl: LC

##### Notes

Flight period: II May - I October

In the study area, the species is found mainly in the lowlands and hills, up to ca. 475 m a.s.l. At these elevations, *O.brunneum* is rather widespread throughout the three provinces in a broad variety of lotic habitats. Records between 600 and ca. 1250 m a.s.l. are probably referring to dispersing individuals.

#### Orthetrum cancellatum

(Linnaeus, 1758)

B34440BA-1929-56CE-A1C6-9BF8CEEB6B00

##### Native status

R

##### Conservation status

erl: LC; irl: LC

##### Notes

Flight period: III April - II October

Common and widespread in the study area, especially at low and medium elevations, although dispersing individuals can be found up to ca. 1220 m a.s.l. It occurs in a wide range of habitats, such as small, medium and large lakes, watering ponds, mature marshlands, rivers and ditches.

#### Orthetrum coerulescens

(Fabricius, 1798)

8760E7D9-7976-5127-BA9C-B9093703AE66

##### Native status

R

##### Conservation status

erl: LC; irl: LC

##### Notes

Flight period: III May - II October

Rather widespread in the study area, the species is found in small to medium lakes, ponds, marshlands, fens (where it generally selects slow-flowing water microhabitats) and along rivers, small streams and ditches. It occurs up to ca. 1160 m a.s.l. (Camaggiore fen), though it is more frequent at low elevations.

#### Crocothemis erythraea

(Brullé, 1832)

12ABEEBE-7EFB-59DC-8634-F5A81ABCD22D

##### Native status

R

##### Conservation status

erl: LC; irl: LC

##### Notes

Flight period: III April - III October

Very common in the study area, it is widespread up to ca. 400 m a.s.l., with a few records up to ca. 940 m a.s.l. The species occupies a wide range of lentic and lotic habitats, both mature and newly established also in degraded and rather urbanised areas.

#### Sympetrum danae

(Sulzer, 1776)

B451B882-52C7-5FA4-ADF7-4B03A3966EB9

##### Native status

Re

##### Conservation status

erl: LC; irl: LC

##### Notes

Flight period: III June - III September

The species occurred in the past, until at least the 1970s, at the Piani di Nesso and Pian del Tivano fens (located a few km apart). Despite *ad hoc* research conducted in 2022, that population has to be considered nowadays extinct. In the past, it should have been much more abundant and occurring at lower elevations, as was the case in Trentino (Assandri 2019, Assandri 2020). Two records in the Pian di Spagna and Lake Mezzola area (27/06/1985 and 17/07/1986), one at Lake Pusiano (21/09/1972) (Balestrazzi and Pavesi 2008) and one more recent observation (11/08/2015) at Monte Muggio, Casargo LC (ca. 1540 m a.s.l.; G. Bazzi obs., Fig. 3g) are probably attributable to the individual moving from nearby populations.

#### Sympetrum depressiusculum

(Selys, 1841)

17229FF8-050D-5543-81ED-B4C0D15294BD

##### Native status

R

##### Conservation status

erl: VU; irl: EN

##### Notes

Flight period: II June - I November

Rather localised, it occurs along the Adda River, at Lake Alserio and Lake Pusiano and in the heathlands and environmental restorations of the south-western part of the study area; however, its stronghold is the Pian di Spagna, where a large population of national importance has been found. Historical records in the northern Lake Como CO-LC and throughout the high Brianza CO-LC suggest that, in the past, the species should have been more widespread, for example, it occurred in the lakeshore marshland area between Dongo and Domaso CO (Morton 1926), which is now reclaimed. It is interesting to point out that historical data hint that, in the past, some individuals emerged as early as May. The species occurs at marshlands, both mature and recently restored, wet meadows and other ephemeral habitats, such as those found in the heathlands. It has been recorded up to ca. 600 m a.s.l., although it is more common at lower elevations, below 300 m a.s.l.

#### Sympetrum fonscolombei

(Selys, 1840)

B160CAFF-C5FE-56ED-9CE8-03CA131D6C14

##### Native status

R

##### Conservation status

erl: LC; irl: LC

##### Notes

Flight period: I May - III November

Common and widespread in the study area, from the lowlands up to ca. 1600 m a.s.l. It occurs in a wide variety of lentic and lotic habitats; migrating individuals can be observed far away from breeding sites, even within urban areas and at mountain grasslands. The apparent absence from the northern Como Province is likely due to lack of data for this area.

#### Sympetrum meridionale

(Selys, 1841)

5D44DA0A-F8D8-5746-A460-FC2D5BD85397

##### Native status

NR

##### Conservation status

erl: LC; irl: LC

##### Notes

Only three recent records of this migratory species in the study area (Parco delle Groane, 21.08.2016, A. Minicò obs.; Lake Pusiano, 13.09.2018, G. Bazzi obs., Fig. 3f; Laghetto di Piona, 28.09.2021, R. Brembilla obs.) could indicate both an incoming northwards expansion following climate change or better coverage of the area compared to the past. To date, there is no evidence of reproduction in the study area.

#### Sympetrum pedemontanum

(Müller in Allioni, 1766)

E890FE4E-A85C-5F6B-B79C-F12FCFC5D928

##### Native status

R?

##### Conservation status

erl: LC; irl: LC

##### Notes

Flight period: I July - I September

It mostly occurs at ephemeral habitats and marshlands in the lowlands; dispersing individuals can be found also at higher elevations (a single record at Camaggiore fen, ca. 1160 m a.s.l.; G. Pirotta obs.). Most of the recent records likely refer to dispersing individuals, although breeding attempts are possible at the southern edge of Monza and Brianza Province, which is close to known breeding sites. Historical records from Pian di Spagna, northern Lake Como, Lake Segrino, Lake Annone, Lake Olginate and Adda River, suggest a much wider distribution in the study area at least until the 1990s. This is in accordance with other evidence elsewhere in Europe and northern Italy, reporting that, before the second half of the 20^th^ century, the species was largely confined to temporary wetlands and wet meadows flooded by melting snow, such as the flood plains of mountain valleys. Subsequently, it almost disappeared together with these habitats and now it mostly occurs in artificial habitats with variable water levels and maintained at early successional stages (Boudot and Kalkman 2015, Assandri 2020).

#### Sympetrum sanguineum

(Müller, 1764)

4D1F5341-B65C-5BBE-A484-AF7D3390FF82

##### Native status

R

##### Conservation status

erl: LC; irl: LC

##### Notes

Flight period: III May - III October

Rather widespread in the study area, especially in the lowlands and hills up to ca. 950 m a.s.l. It is found in marshlands, small and medium lakes and along minor, slow-flowing waterways rich in aquatic vegetation. It tends to avoid highly-urbanised areas, such as large parts of the Monza and Brianza Province. There is some evidence of decline due to habitat loss also for this still common species.

#### Sympetrum striolatum

(Charpentier, 1840)

7A98C1C0-C1ED-5FC1-8F9E-303E1CADC57B

##### Native status

R

##### Conservation status

erl: LC; irl: LC

##### Notes

Flight period: I June - III December

Widespread, it occurs mainly below 400 m a.s.l., with a handful of records of breeding individuals up to ca. 1170 m a.s.l. It occurs at small and medium lakes, ponds, watering puddles, marshlands and slow-flowing portions of rivers and streams. Dispersing individuals can be found far away from suitable breeding habitats.

#### Sympetrum vulgatum

(Linnaeus, 1758)

83A464C1-F507-5CAC-AC2E-37A049D96002

##### Native status

R

##### Conservation status

erl: LC; irl: LC

##### Notes

Flight period: I June - III October

Localised, breeds at Pian di Spagna, Lake Garlate and Lake Olginate (where a huge population occurs) and in the northern part of Parco Adda Nord. The other few observations elsewhere probably refer to dispersing individuals from these areas.

#### Trithemis annulata

(Palisot de Beauvais, 1807)

9100B105-5CEB-5C2D-8BAE-557C08493767

##### Native status

R

##### Conservation status

erl: LC; irl: LC

##### Notes

Flight period: III June - III October

This species colonised the study area in 2019, following a general tendency to expand northwards, which led to the colonisation of Lombardy the year before (Gheza et al. 2019). All the reported records are localised south of Lake Como and below 380 m a.s.l., except for an individual record at Lake Piano (Fig. 3h). Repeated observations at some localities (Lakes Sartirana, Annone and Alserio) suggest that the species have started to breed in the study area.

### Species excluded

#### Chalcolestes parvidens

(Artobolevskii, 1929)

5F26953D-D155-5895-A23A-26D20C6305F9

##### Native status

EX

##### Conservation status

erl: LC; irl: LC

##### Notes

A female *C.parvidens* was reported on 08/09/2017 at Palude di Brivio LC (P. Bonvicini, G. Radaelli obs.). This taxon is hard to separate from *C.viridis* in the field. The photographic documentation obtained on that occasion did not allow for confirmation of the species' identity unambiguously; thus, the taxon was provisionally excluded from the odonatofauna of the study area.

#### Sympetrum flaveolum

(Linnaeus, 1758)

827BA135-8C62-55BA-9105-C4F6FB4D10ED

##### Native status

EX

##### Conservation status

erl: LC; irl: VU

##### Notes

A single, historical record of the species (Capiago Intimiano CO, 10.06.1964; Balestrazzi and Pavesi (2008)) should be considered questionable as entirely out of the known range of the species, which reaches Lombardy only in the Apennines (Riservato et al. 2014a).

## Discussion

Overall, in the Lario and Brianza area, we confirmed the past or present occurrence of 54 odonate species (20 Zygoptera and 34 Anisoptera). Of these, one (*Erythrommanajas*) occurred exclusively before 1978, while seven species (*Lestesbarbarus*, *Coenagrionscitulum*, *Aeshnaaffinis*, *Anaxephippiger*, *Somatochloraarctica*, *Sympetrummeridionale* and *Trithemisannulata*) have been only recorded since 2000 onwards. All those latter species, with the exception of *Somatochloraarctica*, are warm-adapted species which are expanding their range northwards in response to anthropogenic climate warming (Hassall and Thompson 2008, Ott 2010, Gheza et al. 2019, Piretta and Assandri 2019, Termaat et al. 2019, Assandri 2020). Further two species (*Chalcolestesparvidens* and *Sympetrumflaveolum*) were excluded from the fauna of the area, as we consider their occurrence questionable.

Fifty-one species reproduce - or have reproduced in the past - in the study area. Amongst these, two cold-adapted species (*Erythrommanajas* and *Sympetrumdanae*) no longer reproduce; in both cases, the local extinction is imputable to habitat loss, alteration and fragmentation, climate change and, at least for the declining *E.najas*, isolation from the few known nearby populations (Boudot and Kalkman 2015, Assandri 2019, Assandri 2020). On the other hand, *Sympetrumpedemontanum* was a rather diffuse breeder in the past, whereas now its reproduction is to be confirmed. Finally, *C.scitulum*, a southern species which is actively colonising northern Italy (Evangelista 2011, Swaegers et al. 2014, Assandri 2019), is to be considered an irregular breeder in the area. An additional species, *Sympecmapaedisca*, likely reproduce/reproduced in the area, although there is no definite breeding evidence either before nor after 2000. The remaining three species (*Lestesbarbarus*, *Anaxephippiger* and *Sympetrummeridionale*), which to the best of our knowledge do not reproduce in the area, are known to undertake regular dispersive or even migratory movements from their southern breeding sites to the north.

Examining the species of conservation interest, three are considered at risk by the European Red List, i.e. *Cordulegasterbidentata*, *Oxygastracurtisii* (both Near Threatened) and *Sympetrumdepressiusculum* (Vulnerable) (Kalkman et al. 2010). Additionally, seven species are categorised in a risk category of the Italian Red List: *Sympecmapaedisca* (Critically Endangered); *Coenagrionpulchellum* (Near Threatened); *Erythrommanajas* (Endangered); *O.curtisii* (Near threatened); *Corduliaaenea* (Near Threatened); *Somatochloraarctica* (Near Threatened); and *S.depressiusculum* (Endangered) (Riservato et al. 2014b). Two of these species are also European conservation priority species, as they are listed in the Annexes of the 92/43/CEE Habitats Directive, *O.curtisii* in annexes II-IV and *S.paedisca* in annex II.

From a national perspective, the study area appears of primary importance at least for *O.curtisii*, which is found at several sites and locally reaches rather high densities; however, its distribution, especially with regards to Lake Como, where a few breeding sites were found despite the abundance of records referring to maturing individuals, is yet to be fully clarified and further research is needed to delineate its range within this area. Another species of conservation priority, *S.depressiusculum* has, in the Lario area, one of the most important strongholds of the whole Italian territory. The Pian di Spagna wet meadows, indeed, support a large population of this species, with up to ca. 1,500 individuals recorded at the same time. Additionally, the localised and conservation priority *S.paedisca* reaches, in the study area, the eastern limit of its current Italian range. The recently-discovered locality reported in the present study adds to the few known sites of occurrence of the species for Lombardy, where, until 2014, it was deemed extinct and then rediscovered (Canovi et al. 2014, Riservato et al. 2014a, Gheza 2016). The area further hosts two distinct *Sympetrumvulgatum* populations. In Italy, *S.vulgatum* is mainly confined to the Alps (Riservato et al. 2014a); thus, this lowland, flourishing population (with up to 100 individuals observed at the same time), located at the gateway to the Po Plain, represents an exception.

This review of the odonatofauna of the area across a long study period, also admitting the limited availability of historical data, allowed us to highlight that the odonates of Lario and Brianza are dealing with several conservation issues. Various species disappeared from several sites between the pre- and post-2000 periods and one, *E.najas*, has become extinct from the odonatofauna of the area. The most impacted were the species inhabiting wet meadows and well-preserved, mature wetlands, habitats which were diffusely reclaimed, reduced and altered in the last decades to leave space for human activities. Indeed, while to most of the lakes and rivers were accorded some kind of protection (mainly in the form of Regional Parks or by including them in the Natura 2000 network), ephemeral freshwater habitats and wetlands were often overlooked (Bazzi et al. 2020). The decline and local range contraction of some of these species mirror those acting at a broader level, as in the case of *E.najas*, *S.depressiusculum* (Riservato et al. 2014b) and *S.pedemontanum* (Boudot and Kalkman 2015, Assandri 2020). For other species, such as *Sympecmafusca* and *Somatochloraflavomaculata*, whose populations are stable at the national level (Riservato et al. 2014b), the downfall seems entirely attributable to the uncontrolled growth of urban and industrial areas and infrastructure at the expenses of natural habitats. It is worth pointing out that some of these local extinctions involved parks, reserves and SCIs (e.g. Riserva Naturale del Pian di Spagna e del Lago di Mezzola, Riserva Naturale Lago di Piano, Parco dell’Adda Nord), suggesting that legal protection alone is not enough if it does not involve a real effort to keep the key habitats unspoiled. In fact, most lake shores are incorporated into the urban matrix and isolated from the nearby marshlands, which, in turn, are, at best, fragmented, when not completely reclaimed. The same holds true for the remnant of heathlands and their associated ephemeral wetlands (which harbour relevant population of *Lestessponsa*, *Lestesvirens*, *Aeshnaaffinis* and possibly *Sympecmapaedisca*), which are now interspersed by urban and industrial areas and other infrastructure and for wetlands along rivers and floodplains, which were often also drained and deprived of the water sources that once fed them. In this sense, the most affected area is certainly the Province of Monza and Brianza, densely populated and, especially in the central and western part, almost fully anthropogenised.

However, due to its geographic location and abundance of heterogenous freshwater habitats and despite heavy urbanisation, Lario and Brianza still harbour a notable odonate diversity. Indeed, the 54 confirmed species represent more than a half (57%) of the 95 species recorded in Italy (La Porta et al. 2023) and 37% of the 146 recorded in Europe (Boudot and Kalkman 2015, Dijkstra et al. 2023).

In the years to come, further efforts should be focused on understanding how and to what extent odonate communities are affected by human activities in this area. Further research is also needed to clarify the range of some species (e.g. *O.curtisii*) and to investigate sites that are yet to be fully explored, such as the mountainous part of Como Province. From a conservation perspective, it should be noted that several key sites are still not protected. It is the case of both the main fens of the area (Piani di Nesso and Camaggiore), as well as of Lake Annone and La Poncia area, which would deserve special attention due to their importance to odonates and other taxa.

In Italy, since the publication of the first provisional odonate distribution atlas (Riservato et al. 2014a), efforts to fill the ‘Wallacean shortfall’ (Lomolino 2004) also for this taxonomic group have steadily increased (e.g. Sindaco et al. (2018), Assandri (2019), Corso et al. (2019), Dal Cortivo and Roncen (2019), Bonometto (2020), Zandigiacomo et al. (2020)). This contribution follows this red line providing the first large synthesis on the odonate fauna of one of the more densely inhabited areas of the Country, which, until now, has been surprisingly overlooked and neglected by odonatologists. We hope that this catalogue could constitute a solid basis for other studies and provide information for freshwater conservation actions and landscape planning in the Lario and Brianza Regions.

## Supplementary Material

XML Treatment for Calopteryx splendens

XML Treatment for Calopteryx virgo

XML Treatment for Sympecma fusca

XML Treatment for Sympecma paedisca

XML Treatment for Chalcolestes viridis

XML Treatment for Lestes barbarus

XML Treatment for Lestes sponsa

XML Treatment for Lestes virens

XML Treatment for Platycnemis pennipes

XML Treatment for Ischnura elegans

XML Treatment for Ischnura pumilio

XML Treatment for Enallagma cyathigerum

XML Treatment for Pyrrhosoma nymphula

XML Treatment for Coenagrion puella

XML Treatment for Coenagrion pulchellum

XML Treatment for Coenagrion scitulum

XML Treatment for Erythromma lindenii

XML Treatment for Erythromma najas

XML Treatment for Erythromma viridulum

XML Treatment for Ceriagrion tenellum

XML Treatment for Aeshna affinis

XML Treatment for Aeshna cyanea

XML Treatment for Aeshna isoceles

XML Treatment for Aeshna juncea

XML Treatment for Aeshna mixta

XML Treatment for Anax imperator

XML Treatment for Anax parthenope

XML Treatment for Anax ephippiger

XML Treatment for Gomphus vulgatissimus

XML Treatment for Onychogomphus forcipatus

XML Treatment for Cordulegaster bidentata

XML Treatment for Cordulegaster boltonii

XML Treatment for Cordulia aenea

XML Treatment for Somatochlora arctica

XML Treatment for Somatochlora flavomaculata

XML Treatment for Somatochlora metallica

XML Treatment for Oxygastra curtisii

XML Treatment for Libellula depressa

XML Treatment for Libellula fulva

XML Treatment for Libellula quadrimaculata

XML Treatment for Orthetrum albistylum

XML Treatment for Orthetrum brunneum

XML Treatment for Orthetrum cancellatum

XML Treatment for Orthetrum coerulescens

XML Treatment for Crocothemis erythraea

XML Treatment for Sympetrum danae

XML Treatment for Sympetrum depressiusculum

XML Treatment for Sympetrum fonscolombei

XML Treatment for Sympetrum meridionale

XML Treatment for Sympetrum pedemontanum

XML Treatment for Sympetrum sanguineum

XML Treatment for Sympetrum striolatum

XML Treatment for Sympetrum vulgatum

XML Treatment for Trithemis annulata

XML Treatment for Chalcolestes parvidens

XML Treatment for Sympetrum flaveolum

08D3F8D0-E1CB-58A8-9337-DEF12405D1FB 10.3897/BDJ.11.e111358.suppl1 Supplementary material 1 Distribution maps of the odonates of Lario and Brianza Data type Images File: oo_880269.pdf https://binary.pensoft.net/file/880269 Gaia Bazzi, Giacomo Assandri

533975EE-224E-52D0-B0C5-CE7E8A748DC7 10.3897/BDJ.11.e111358.suppl2 Supplementary material 2 Site-specific checklist of the Odonata of Natura 2000 network of Lario and Brianza Data type Table File: oo_885715.pdf https://binary.pensoft.net/file/885715 Gaia Bazzi, Giacomo Assandri

F0C9FCDE-2C7B-5770-A522-891EB6CA5DAA 10.3897/BDJ.11.e111358.suppl3 Supplementary material 3 Site-specific checklist of the Odonata of the protected areas of Lario and Brianza Data type Table File: oo_885716.pdf https://binary.pensoft.net/file/885716 Gaia Bazzi, Giacomo Assandri
