# Supplementary material for: Odonate diversity of a highly urbanised region: An annotated checklist of the damselflies and dragonflies (Insecta, Odonata) of Lario and Brianza (Lombardy, N Italy)
Source: Biodivers Data J. 2023 Nov 7;11:e111358. doi: 10.3897/BDJ.11.e111358 (PMC10646535; doi:10.3897/BDJ.11.e111358)

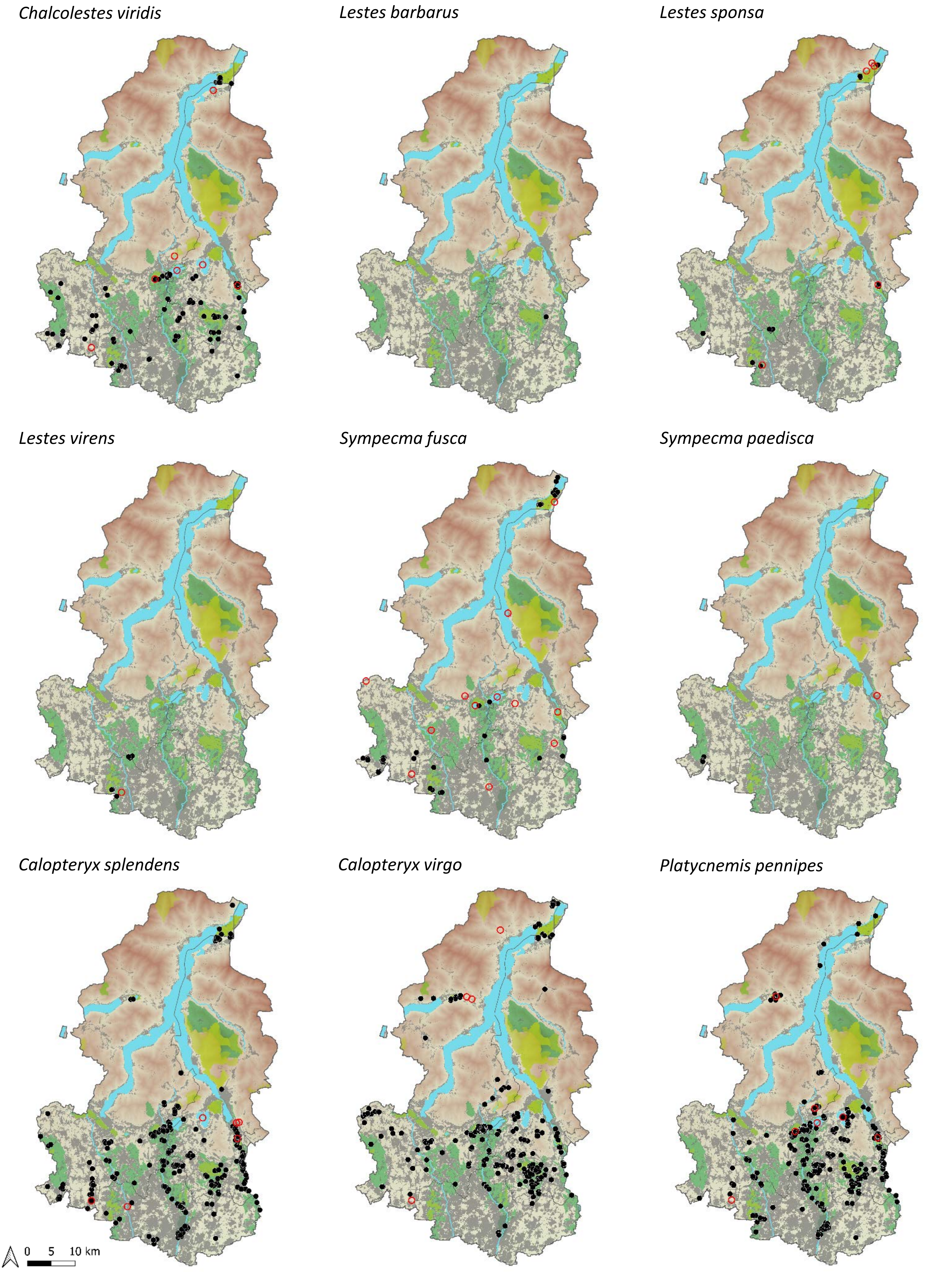
Chalcolestes viridis
Lestes barbarus
Lestes sponsa
Lestes virens
Sympecma fusca
Sympecma paedisca
Calopteryx splendens
Calopteryx virgo
Platycnemis pennipes
0 5 10 km

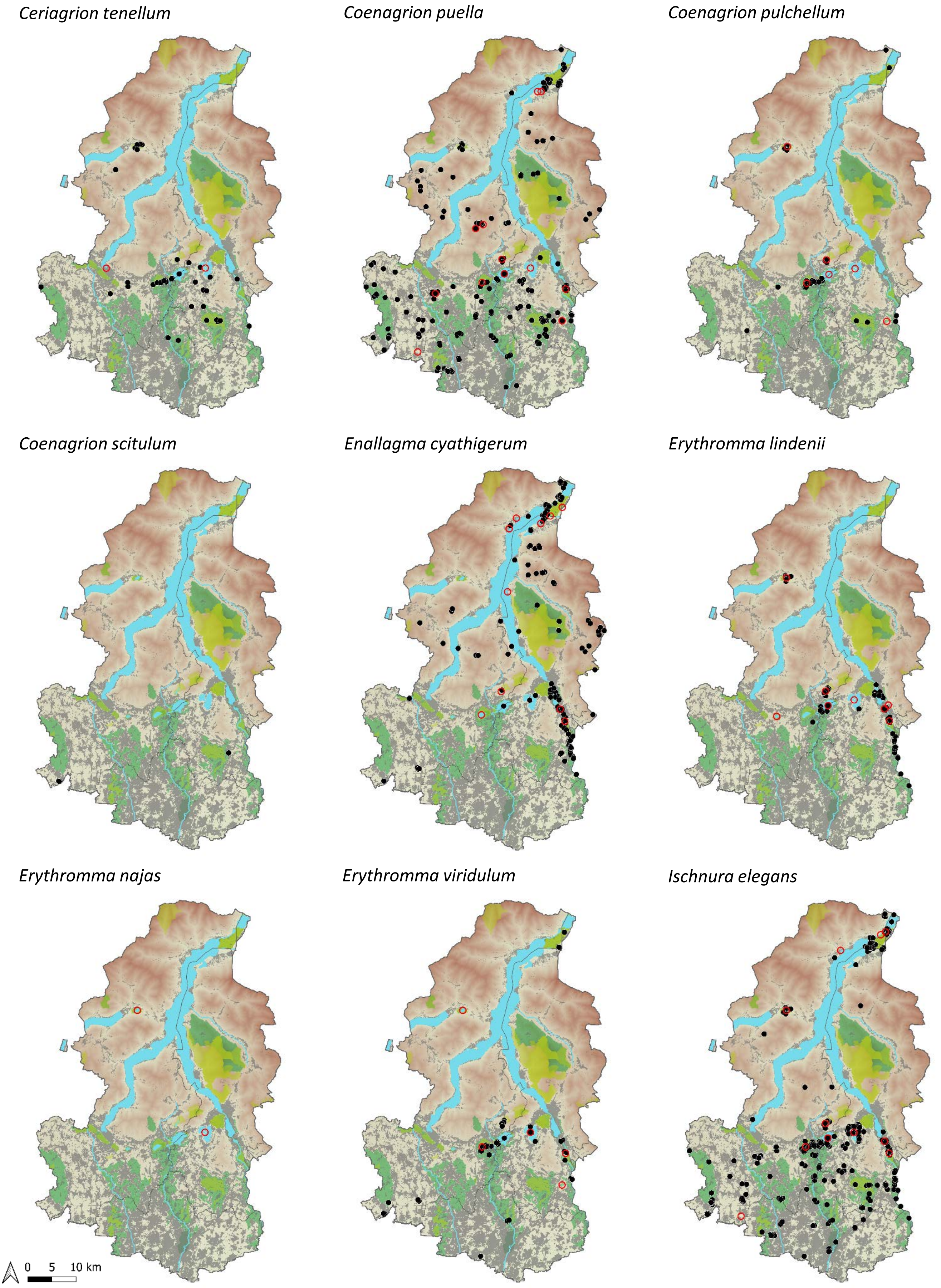
Ceriagrion tenellum
Coenagrion puella
Coenagrion pulchellum
Coenagrion scitulum
Enallagma cyathigerum
Erythromma lindenii
Erythromma najas
Erythromma viridulum
Ischnura elegans
0 5 10 km

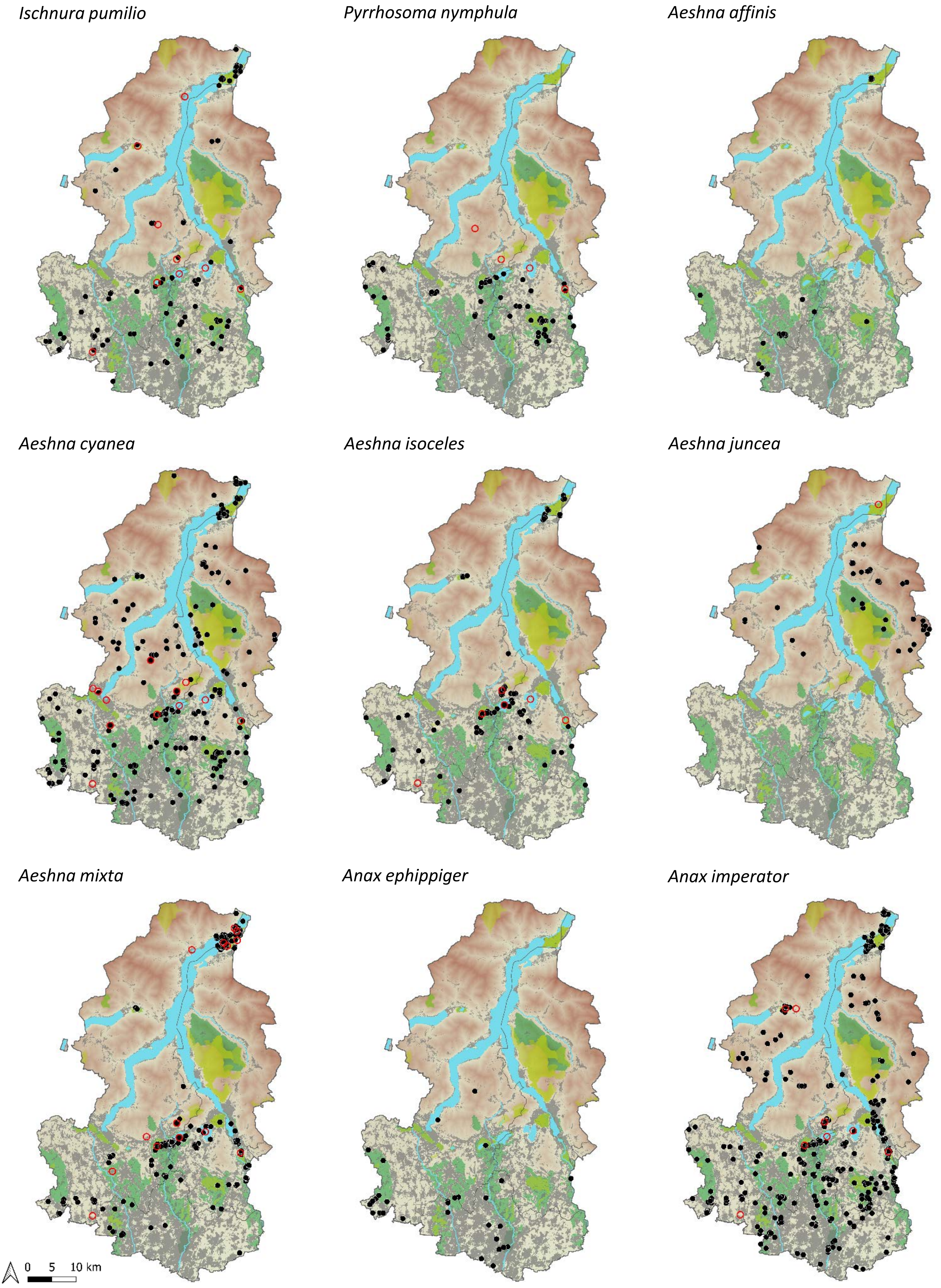
Ischnura pumilio
Pyrrhosoma nymphula
Aeshna affinis
Aeshna cyanea
Aeshna isoceles
Aeshna juncea
Aeshna mixta
Anax ephippiger
Anax imperator
0 5 10 km

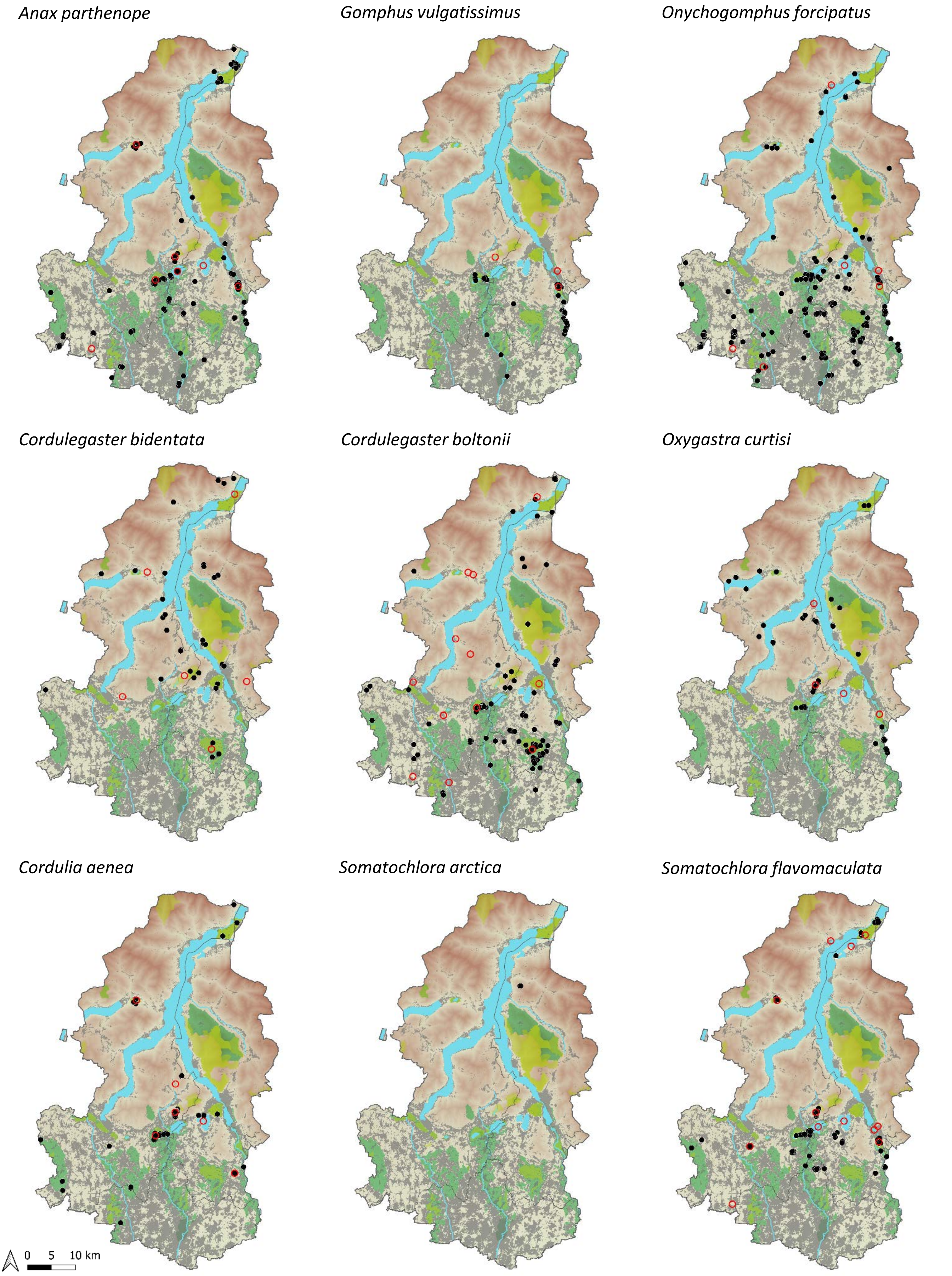
Anax parthenope
Gomphus vulgatissimus
Onychogomphus forcipatus
Cordulegaster bidentata
Cordulegaster boltonii
Oxygastra curtisi
Cordulia aenea
Somatochlora arctica
Somatochlora flavomaculata
0 5 10 km

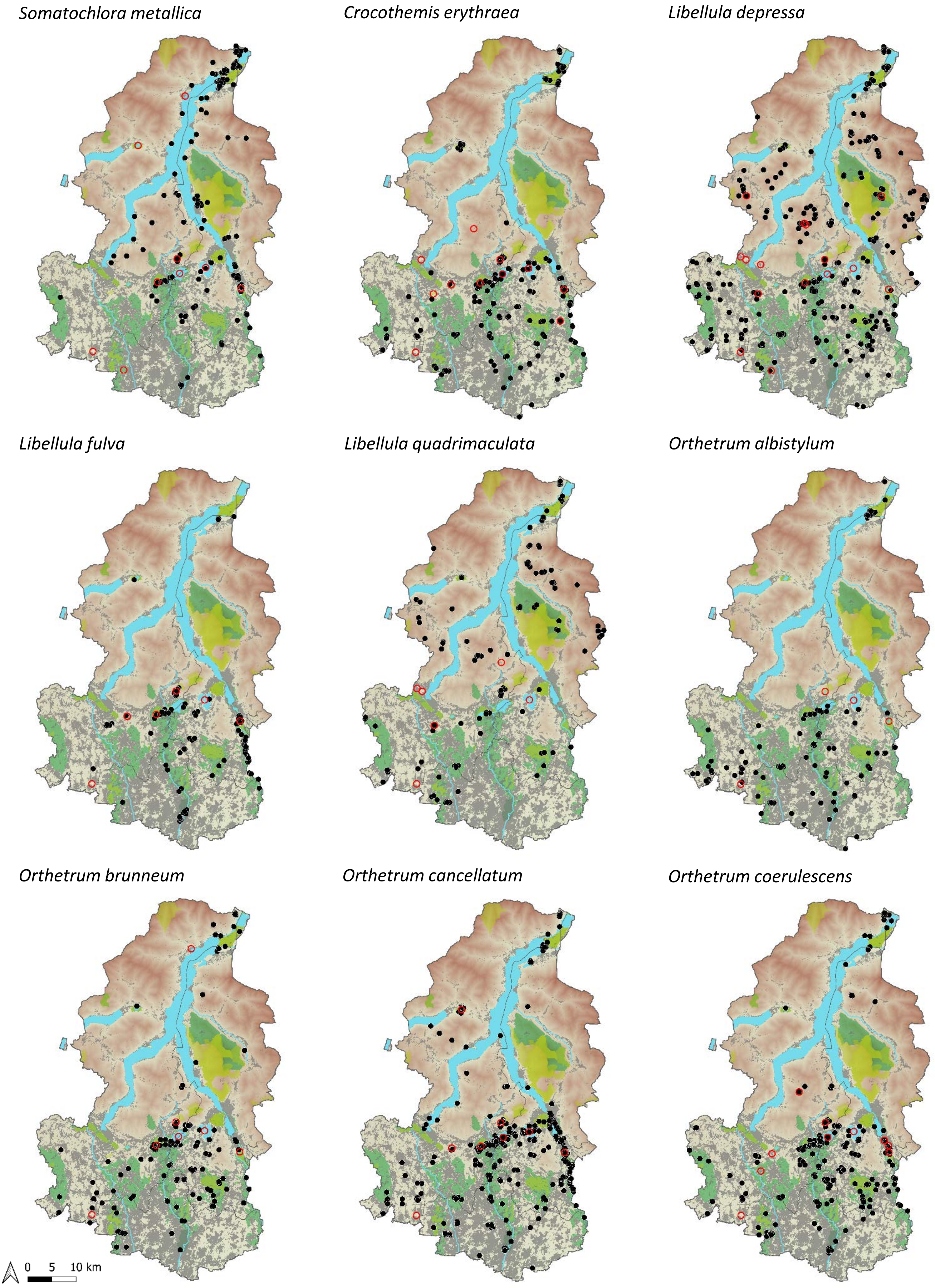

Somatochlora metallica
Crocothemis erythraea
Libellula depressa
Libellula fulva
Libellula quadrimaculata
Orthetrum albistylum
Orthetrum brunneum
Orthetrum cancellatum
Orthetrum coerulescens
0 5 10 km

Sympetrum danae

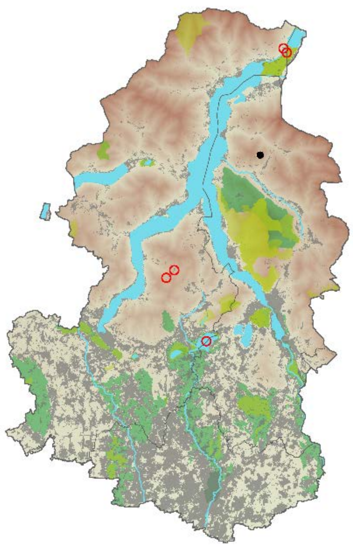

Sympetrum depressiusculum

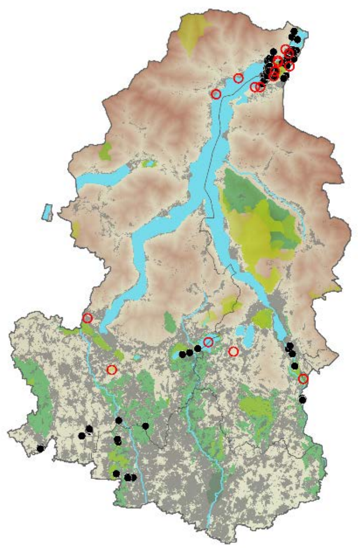

Sympetrum fonscolombii

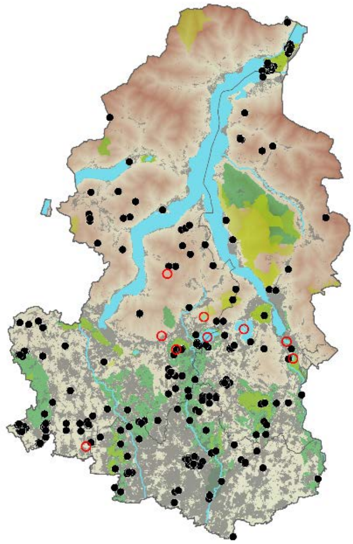

Sympetrum meridionale

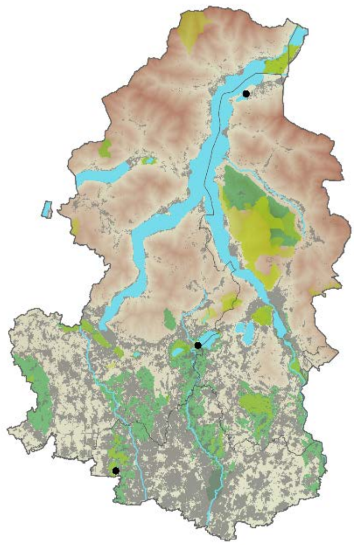

Sympetrum pedemontanum

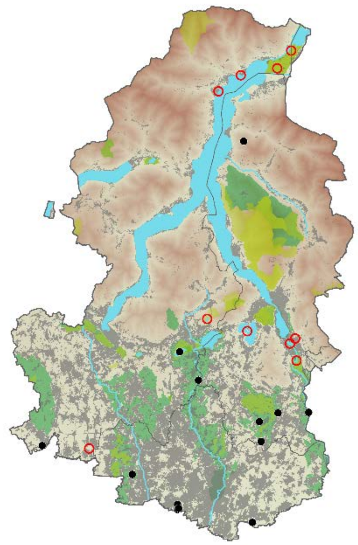

Sympetrum sanguineum

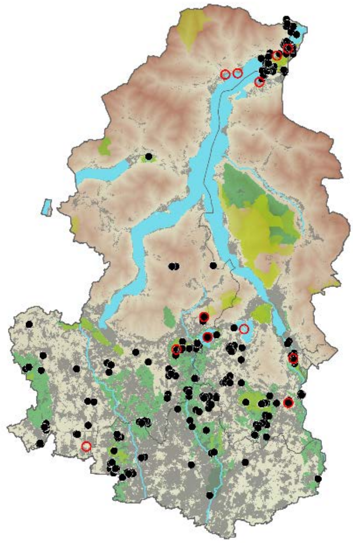

Sympetrum striolatum

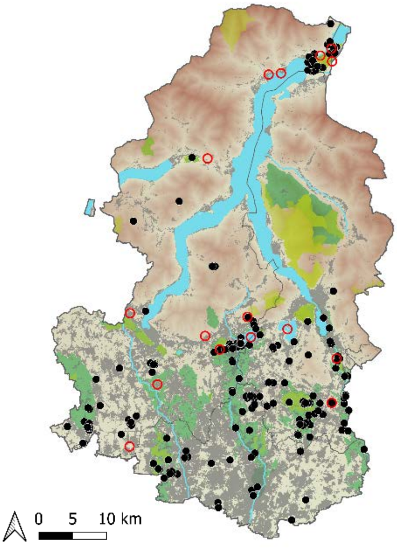

Sympetrum vulgatum

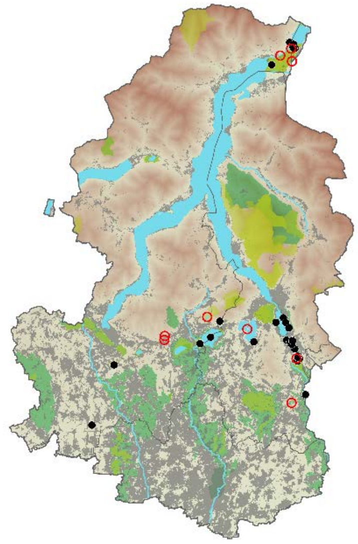

Trithemis annulata

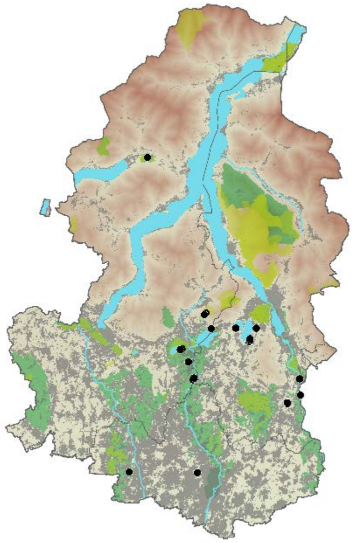

Supplement: Supplementary material 1 — Distribution maps of the odonates of Lario and Brianza [file bdj-11-e111358-s001.pdf]
