# Supplementary material for: Odonate diversity of a highly urbanised region: An annotated checklist of the damselflies and dragonflies (Insecta, Odonata) of Lario and Brianza (Lombardy, N Italy)
Source: Biodivers Data J. 2023 Nov 7;11:e111358. doi: 10.3897/BDJ.11.e111358 (PMC10646535; doi:10.3897/BDJ.11.e111358)
Supplement: Supplementary material 2 — Site-specific checklist of the Odonata of Natura 2000 network of Lario and Brianza [file bdj-11-e111358-s002.pdf]

Supplementary material 2. Odonate site-specific check-list of the Natura 2000 network of Lario and Brianza

Species names are abbreviated with the first three letters of genus and species. ● = post-2000 only; ○ = pre-2000 only; ◉ = both pre- and post-2000

	Type	Chavir	Lesspo	Lesvir	Symfus	Calspl	Calvir	Plapen	Certen	Coepue	Coepul	Enacya	Erylin	Erynai	Eryvir	Iscele	Iscpum	Pyrnym	Aesaff	Aescya	Aesiso	Aesjun	Aesmix	Anaeph	Anaimp	Anapar	Gomvul	Onyfor	Corbid	Corbol	Oxycur	Coraen	Somfla	Sommet	Croery	Libdep	Libful	Libqua	Ortalb	Ortbru	Ortcan	Ortcoe	Symdan	Symdep	Symfon	Symmer	Symped	Symsan	Symstr	Symvul
Boschi delle Groane	ZSC	●	●	●	●	●		●		●						●			●	●	●		●		●			●							●	●		●	●	●	●	●		●	●	●		●	●	
Fontana del Guercio	ZSC					●	●	●		●								●		●								●		●															●			●		
Grigna Meridionale	ZSC																			●									●	●	●			●																
Grigna Settentrionale	ZSC																					●														◉														
Lago del Segrino	ZSC	○				●		◉	●	◉	◉	◉	◉		●	◉	◉	○		◉	◉		◉		◉	◉	○	●			◉	◉	◉	◉	◉	◉	◉	●	○	◉	◉	◉			○		○	◉	◉	○
Lago di Alserio	ZSC	◉			◉	●	●	◉	●	◉	◉	○			◉	◉	◉	●		◉	◉		◉	●	◉	◉	●	●		◉	●	◉	●	◉	◉	◉	◉	●	●	◉	◉	●		●	◉		●	◉	◉	
Lago di Montorfano	ZSC							●	●	●	●		○			●																			◉		◉				◉							●		
Lago di Olginate	ZSC					●	●	●	●			◉	◉		●	◉				●			●		●	●		●					○	●	●				●	●	●	◉		●	◉		○	●	●	●
Lago di Piano	ZSC					●	●	◉	●	●	◉		◉	○	○	◉	◉			●	●		●		◉	◉		●	●		●	◉	◉	○	●	●	●	●		●	◉	●						●	●	
Lago di Pusiano	ZSC	◉			◉	●	●	◉	●	◉	◉	●	◉		●	◉	◉	●		◉	◉		◉		◉	◉		●		●		●	◉	◉	◉	◉	●		●	◉	◉	◉	○	◉	◉	●		◉	◉	●
Lago di Sartirana	ZSC				○		●	●		◉	○	●			○	●				●	●		●		●	●					●	◉	●	●	◉	●			●		●	●			●			◉	◉	○
Monte Barro	ZSC									●										●			●		●				●	○		●		●	●	●		●			●				●				●	
Palude di Albate	ZSC	●				●	●	●	●	◉						●	●	●		◉	●		●		●	●						●	◉		○	◉	●	◉	●		●	●		○	●			●	●	●
Palude di Brivio	ZSC	●	◉		○	◉	●	◉		◉		◉	◉		◉	◉	◉	◉		◉	◉		◉		◉	◉	◉	○			○		◉	◉	◉	◉	◉		○	○	◉	◉		◉	○		○	◉	◉	◉
P. di Spagna e L. di Mezzola	ZSC	●	◉		●	●	●	●		●	●	◉			●	◉	●		●	●	●	○	◉		●	●		●	○		●	●	◉	●	●	●		●	●	●	●	●	○	◉	●		○	◉	◉	◉
Pineta Ped. di Appiano Gentile	ZSC																																																	
Sasso Malascarpa	ZSC																			●									●	●															●					
Spina Verde	ZSC											●																		●																				
Valle del Dosso	ZSC																			●																														
Valle del Rio Pegorino	ZSC					●				●																																			●					
Valle S. Croce e V. del Curone	ZSC	●				●	●	●	●	●	●					●	●	●	●	●	●		●		●			●	◉	◉					●	●	●	●	●	●	●	●			●			●	●	
Costa del Pallio	ZPS											●																								●														
Grigne	ZPS									●		●								●		●			●				●	●	●			●		◉		●		●										
Il Toffo	ZPS	●			●	●	●	●		●	●	●	●		●	●		●			●		●	●	●	●	●	●			●	●	●	●	●	●	●	●	●	●	●	●		●	●			●	●	●
L. di Mezzola e P. di Spagna	ZPS	●	◉		●	●	●	●		●	●	◉			●	◉	●		●	●	●	○	◉		●	●		●	○		●	●	◉	●	●	●		●	●	●	●	●	○	◉	●		○	◉	◉	◉
Monte Barro	ZPS									●										●			●		●				●	○		●		●		●		●			●				●				●	
Monte Generoso	ZPS																																																	
Monte Resegone	ZPS																																																	
Triangolo Lariano	ZPS																			●									●	●															●					
Valsolda	ZPS																																																	
