# Supplementary material for: Odonate diversity of a highly urbanised region: An annotated checklist of the damselflies and dragonflies (Insecta, Odonata) of Lario and Brianza (Lombardy, N Italy)
Source: Biodivers Data J. 2023 Nov 7;11:e111358. doi: 10.3897/BDJ.11.e111358 (PMC10646535; doi:10.3897/BDJ.11.e111358)
Supplement: Supplementary material 3 — Site-specific checklist of the Odonata of the protected areas of Lario and Brianza [file bdj-11-e111358-s003.pdf]

Supplementary material 3. Odonate site-specific check-list of the protected areas of Lario and Brianza

Species names are abbreviated with the first three letters of genus and species. RP = Regional Park; RR = Regional Reserve. ● = post-2000 only; ○ = pre-2000 only; ◉ = both pre- and post-2000.

	Type	Chavir	Lesbar	Lesspo	Lesvir	Symfus	Sympae	Calspl	Calvir	Plapen	Certen	Coepue	Cuepul	Coesci	Enacya	Erylin	Erynai	Eryvir	Iscele	Iscpum	Pyrnym	Aesaff	Aescya	Aesiso	Aesjun	Aesmix	Anaeph	Anaimp
P. del Monte Barro	RP											●											●			●		●
P. della Grigna Settentrionale	RP											●			●								●		●			●
P. Pineta di Appiano Gent. e Tradate	RP	●		●		●	●	●	●		●	●						●	●	●	●	●	●	●		●	●	●
P. della Valle del Lambro	RP	◉				◉		●	●	◉	●	◉	◉		◉	◉		◉	◉	◉	●	●	◉	◉		◉	●	◉
P. dell'Adda Nord	RP	◉		◉		◉	○	◉	●	◉	●	◉	●		◉	◉		◉	◉	◉	◉		◉	◉		◉	●	◉
P. delle Groane	RP	●		◉	◉	◉		●	●	●		●							●	●	●	●	●	●		◉	●	●
P. di Montevecchia e V. del Curone	RP	●	●			●		●	●	●	●	●	●	●					●	●	●	●	●	●		●		●
P. Spina Verde	RP														●													
Fontana del Guercio	RR							●	●	●		●									●		●					
Lago di Montorfano	RR									●	●	●	●			○			●									
Lago di Piano	RR							●	●	◉	●	●	◉			◉	○	○	◉	◉			●	●		●		◉
Lago di Sartirana	RR					○			●	●		◉	○		●			○	●				●	●		●		●
Pian di Spagna - Lago di Mezzola	RR	●		◉		◉		●	●	●		●	●		◉			●	◉	●		●	●	●	○	◉		●
Riva orientale del lago di Alserio	RR	●				●		●	●	●	●	●	●					●	●	●	●		●	●		●	●	●
Sasso Malascarpa	RR																											
Valle Bova	RR																											
Valsolda	RR																											

	Type	Anapar	Gomvul	Onyfor	Corbid	Corbol	Oxycur	Coraen	Somfla	Sommet	Croery	Libdep	Libful	Libqua	Ortalb	Ortbru	Ortcan	Ortcoe	Symdan	Symdep	Symfon	Symmer	Symped	Symsan	Symstr	Symvul	Triann
P. del Monte Barro	RP				●	○		●		●	●	●		●			●				●				●		
P. della Grigna Settentrionale	RP						●					◉		●													
P. Pineta di Appiano Gent. e Tradate	RP	●		●		●		●	●	●	●	●		●	●	●	●	●			●			●	●		
P. della Valle del Lambro	RP	◉	●	●		◉	●	◉	◉	◉	◉	◉	◉	●	●	◉	◉	◉	○	◉	◉	●	●	◉	◉	●	●
P. dell'Adda Nord	RP	◉	◉	◉		●	◉	●	◉	◉	◉	◉	◉	●	◉	◉	◉	◉		◉	◉		◉	◉	◉	◉	●
P. delle Groane	RP	●		◉		●		●		○	●	◉	●	●	●	●	●	◉		●	●	●	●	●	●		●
P. di Montevecchia e V. del Curone	RP			●	◉	◉				●	●	●	●	●	●	●	●	●			●		●	●	●		
P. Spina Verde	RP					●																					
Fontana del Guercio	RR			●		●															●			●			
Lago di Montorfano	RR										◉		◉				◉							●			
Lago di Piano	RR	◉		●	●		●	◉	◉	○	●	●	●	●		●	◉	●						●	●		●
Lago di Sartirana	RR	●					●	◉	●	●	◉	●			●		●	●			●			◉	◉	○	●
Pian di Spagna - Lago di Mezzola	RR	●		●	○		●	●	◉	●	●	●		●	●	●	●	●	○	◉	●		○	◉	◉	◉	
Riva orientale del lago di Alserio	RR	●	●	●		●	●	●	●	●	●	●	●	●	●	●	●	●		●	●			●	●		●
Sasso Malascarpa	RR				●																●						
Valle Bova	RR																										
Valsolda	RR																										
